# The Angiotensin Metabolite His-Leu Is a Strong Copper Chelator Forming Highly Redox Active Species

**DOI:** 10.1021/acs.inorgchem.4c01640

**Published:** 2024-06-15

**Authors:** Nina E. Wezynfeld, Dobromiła Sudzik, Aleksandra Tobolska, Katerina Makarova, Ewelina Stefaniak, Tomasz Frączyk, Urszula E. Wawrzyniak, Wojciech Bal

**Affiliations:** 1 Chair of Medical Biotechnology, Faculty of Chemistry, Warsaw University of Technology, Noakowskiego 3, 00-664 Warsaw, Poland; 2 Institute of Biochemistry and Biophysics, Polish Academy of Sciences, Pawińskiego 5a, 02-106 Warsaw, Poland; 3 Department of Organic and Physical Chemistry, Faculty of Pharmacy, Medical University of Warsaw, Żwirki i Wigury 61, 02-091 Warsaw, Poland; 4 National Heart and Lung Institute, Imperial College London, Molecular Sciences Research Hub, London W12 0BZ, United Kingdom

## Abstract

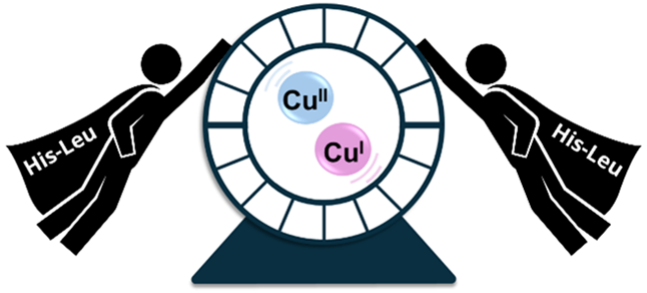

His-Leu is a hydrolytic byproduct of angiotensin metabolism, whose concentration in the bloodstream could be at least micromolar. This encouraged us to investigate its Cu(II) binding properties and the concomitant redox reactivity. The Cu(II) binding constants were derived from isothermal titration calorimetry and potentiometry, while identities and structures of complexes were obtained from ultraviolet–visible, circular dichroism, and room-temperature electronic paramagnetic resonance spectroscopies. Four types of Cu(II)/His-Leu complexes were detected. The histamine-like complexes prevail at low pH. At neutral and mildly alkaline pH and low Cu(II):His-Leu ratios, they are superseded by diglycine-like complexes involving the deprotonated peptide nitrogen. At His-Leu:Cu(II) ratios of ≥2, bis-complexes are formed instead. Above pH 10.5, a diglycine-like complex containing the equatorially coordinated hydroxyl group predominates at all ratios tested. Cu(II)/His-Leu complexes are also strongly redox active, as demonstrated by voltammetric studies and the ascorbate oxidation assay. Finally, numeric competition simulations with human serum albumin, glycyl-histydyl-lysine, and histidine revealed that His-Leu might be a part of the low-molecular weight Cu(II) pool in blood if its abundance is >10 μM. These results yield further questions, such as the biological relevance of ternary complexes containing His-Leu.

## Abstract 2

Coordination properties of dipeptide His-Leu, an angiotensin metabolite, qualify it as a putative low-molecular weight Cu(II) carrier in blood due to the formation of highly stable Cu(II) bis-complexes. These complexes are highly redox active through the Cu(II)/Cu(I) cycle, which may contribute to their biological activity.

## Introduction

The hydrolytic digestion of proteins plays a crucial role in maintaining homeostasis, as part of the more general autophagy process.^1^ The excessive or aged (damaged, e.g., oxidatively) protein molecules are substrates for a large set of proteases that cleave them, presumably down to amino acids, which are then recycled as building blocks of new proteins or metabolized in different ways.^2^ Proteolysis is also used in processes of protein or peptide maturation, in which one or more peptide bonds are cleaved to release the functional molecule in a time- and space-controlled fashion.^3^ These processes occur both intra- and extracellularly, e.g., in the bloodstream. It is not really known, however, whether oligopeptidic intermediates of these processes may have additional functions. The participation in metal ion trafficking is one such possibility, especially for oligopeptides containing Cys, His, Glu, or Asp residues. The angiotensin biosynthesis is a good example of such a process. It starts with angiotensinogen, the medium-sized protein (453 amino acids) released largely from liver into the blood serum.^4,5^ Angiotensinogen participates in the transport of fatty acids and steroid hormones in blood but also serves as a substrate for renin, a protease released from kidney. Renin cuts the angiotensinogen molecule into the N-terminal decapeptide, which constitutes inactive angiotensin I (A1) and the remainder, dubbed des-angiotensinogen. Next, in the lung and kidney blood vessels, A1 is processed by the angiotensin-converting enzyme (ACE) into active octapeptide angiotensin II (A2) by cutting off C-terminal dipeptide His-Leu.^6^ A2 is a very potent direct vasoconstrictor and also stimulates vasopressin release. As such, it is a key endogenous agent that increases blood pressure, even recently approved as a drug for the treatment of vasodilatory shock.^7^ On the contrary, A2 and ACE are among targets of vast research effort in combating hypertension.^8^ However, very little attention was devoted to des-angiotensinogen, which circulates in the bloodstream in significant amounts and exhibits antiangiogenic activity^9,10^ (no direct data are available on des-angiotensinogen concentration, but the steady-state concentration of angiotensinogen is ∼1 μM in healthy humans).^11^ Furthermore, until very recently, no research was devoted to the biological properties of His-Leu, the dipeptide co-released with A2, which may be present in the human bloodstream in large amounts, due to a high rate of A2 turnover. The half-life of A2 is <1 min,^12^ and its steady-state concentration in healthy subjects is in the range of 15–20 pM, but with a large variability, reaching 100 pM in kidney disease patients.^13,14^ Much higher A2 levels are noted in individual tissues, due to receptor binding that slows the turnover.^15^ A crude calculation then provides an estimate of the daily production of His-Leu as 15 μmol (4 mg), which corresponds to the average bloodstream concentration of 3 μM. This speculation is supported by a recent discovery that the blood level of angiotensin1–12, a rarely investigated A2 precursor, is 100-fold higher than that of A2.^16^ The rate of His-Leu digestion to amino acids and/or excretion is not known, but when blood plasma concentrations of His and Leu, approximately 70–120 and 100–200 μM, respectively,^17,18^ are taken into account, strong product inhibition of His-Leu hydrolysis should be expected. With these facts, one can make a micromolar or higher His-Leu range in the bloodstream as large as plausible. His-Leu may be particularly highly concentrated in the lungs and kidneys, the major sites of its production.

The presence of the His residue near the N-terminus of the peptide or protein contributes to its high Cu(II) affinity. The biologically relevant examples include sequences containing His at position 3 (His-3) like in human serum albumin (HSA),^19^ the extracellular domain of copper transporter Ctr1^20,21^ or the N-truncated Aβ form, Aβ_4–*x*_.^22^ Those containing His at position 2 (His-2) are present in wound healing factor GHK,^23^ yeast α-factor,^24^ insulin-related GHTD-NH_2_^25^ peptide, and many others.^26^ With His at position 1 (His-1), the His-Leu peptide could also be a strong Cu(II) chelator, serving as a low-molecular weight (LMW) ligand in the exchangeable Cu(II) pool in the blood. The composition of the LMW Cu(II) pool is still a matter of extensive scientific debate, but with the His-2 peptide GHK and the His amino acid recognized as the main candidates for such a role,^27,28^ the contribution of His-1 peptide His-Leu is also possible. Unfortunately, it is hard to verify this hypothesis due to inconsistencies in literature data on Cu(II) coordination of the His-1 dipeptides.

More than a dozen of studies were devoted to Cu(II) binding by the His-1-containing His-Xaa dipeptides, where Xaa was Gly,^29−39^ Ala,^31,40^ Val,^40^ Met,^41^ Phe,^42^ Tyr,^42^ and Lys.^43,44^ Potentiometric titrations were used as the main experimental method in most of these papers, with auxiliary spectroscopic studies, except of the electronic paramagnetic resonance (EPR) study of the Cu(II)/His-Gly^31,38^ and Cu(II)/His-Lys systems.^43,44^ Most researchers detected mono- and bis-complexes with various degrees of deprotonation, depending on the pH. In many cases, the 2:2 dimers were also indicated at higher pH values. His-Leu complexes were studied in the context of DNA cleavage by ternary complexes of His-Xaa dipeptides with histamine and ethylenediamine.^45^ An overly simplistic coordination model, assuming only 1:1 Cu(II)/His-Xaa complexes, was proposed. This excludes the possibility of comparing these results with others.

A recent study investigated the physiological effects of His-Leu in rats.^46^ No direct association was found between His-Leu administration and metal ion (including copper) distribution in lung tissue. However, the levels of A2 precursors in humans are generally 10-fold higher than in rodents.^47^ The level of His-Leu has not been determined in human blood, but when a very high rate of conversion of A1 into A2 is taken into account, even high micromolar His-Leu concentrations in blood serum can be expected. If so, then His-Leu might participate in human (unlike rodent) copper metabolism. In this context, we undertook a systematic study of the Cu(II)/His-Leu system, including stoichiometry, affinity, coordination modes, and the reactivity of the resulting complexes.

## Experimental Methods

### Potentiometry

Potentiometric titrations of the His-Leu peptide and its Cu(II) complexes were performed on a 907 Titrando automatic titrator (Metrohm, Herisau, Switzerland) using a Biotrode combined glass electrode (Metrohm) calibrated daily by nitric acid titrations.^48^ The 100 mM NaOH solution (free of carbon dioxide) was used as a titrant, and 1.5 mL samples were prepared in a 96 mM KNO_3_/4 mM HNO_3_ solution. Complex formation was studied using six different peptide:Cu(II) molar ratios ranging from 1.1 to 2.6. All experiments were performed under argon at 25 °C in the pH range of 2.7–11.5. The obtained data were analyzed using SUPERQUAD and HYPERQUAD.^49,50^

### Ultraviolet–Visible (UV–vis) and Circular Dichroism (CD)

The spectrometric titrations were recorded at 25 °C on a LAMBDA 950 UV–vis–near-infrared (NIR) spectrophotometer (PerkinElmer) over the spectral range of 200–900 nm and on a model J-815 CD spectropolarimeter (Jasco) at 230–800 nm, with 1 cm path length quartz cuvettes (Helma). For pH-metric titrations, samples of 2.75 mM His-Leu with 2.50 mM CuCl_2_ or samples of 5.00 mM His-Leu with 2.50 mM CuCl_2_ were titrated with small amounts of a concentrated NaOH solution, and the spectra were recorded for selected pH values. For His-Leu titrations, a sample of 0.6 mM His-Leu with 0.5 mM CuCl_2_ was titrated at pH 7.4 with small aliquots of an 84 mM His-Leu solution, reaching final His-Leu concentrations of 0.7, 0.8, 0.9, 1.0, 1.25, 1.5, 2.5, 5.0, and 10 mM.

### Electron Paramagnetic Resonance (EPR)

Continuous wave (CW) EPR spectra were recorded on a SPINSCAN X instrument (Adani, Minsk, Belarus) operating in the X-band. The following acquisition parameters were used: time sweep, 180 s; modulation amplitude, 700 μT; power attenuation, 15 db (2.5 mW); and two scans accumulated. The measurements were performed at 24 °C, using an Adani Temperature Control Unit and 50 μL standard tubes. To analyze pH-dependent structural changes of the complexes, the samples containing 5 mM Cu(II) and 6 or 10 mM His-Leu were prepared and their pH values were gradually increased in the range of 3–11.5 using small amounts of a concentrated NaOH solution. To assess the presence of the potential Cu(II)/His-Leu dimer at pH 7.4, the measurements were performed for a series of diluted solutions, 10 mM CuCl_2_ with 12 mM His-Leu, 7.5 mM CuCl_2_ with 9.0 mM His-Leu, 5.0 mM CuCl_2_ with 6.0 mM His-Leu, 2.5 mM CuCl_2_ with 3.0 mM His-Leu, 1.0 mM CuCl_2_ with 1.2 mM His-Leu, and 0.5 mM CuCl_2_ with 0.6 mM His-Leu. Small amounts of NaOH and HCl were applied to adjust the pH values of those samples to 7.4.

Spectral simulations were performed using the EasySpin toolbox for Matlab R2017a. For all spectral simulations, the “chili” function was used.^51^ Spectral optimization was performed using the “esfit” function.^52^

### Isothermal Titration Calorimetry (ITC)

Titrations were carried out on a Nano ITC standard-volume calorimeter (TA Instruments, New Castle, DE). The sample cell (950 μL) was filled with a degassed 0.6–1.8 mM His-Leu solution in 20 mM HEPES and 100 mM KNO_3_ (pH 7.4). The syringe (250 μL) was loaded with degassed 6 mM CuCl_2_ in 100 mM KNO_3_. The final Cu:His-Leu ratios achieved after the last injection were 0.9, 1.4, and 2.7. Typically, 16 μL of a CuCl_2_ solution was added at 1000 s intervals, while it was being stirred at 150 rpm. The measurements were performed at 25 °C. Background titrations were subtracted from each experimental titration. The preliminary estimates of thermodynamic parameters were obtained by using an analytical model implemented in the Origin software package, as described previously.^53^ Finally, the data were analyzed with SEDPHAT version 15.2b using the global-fitting feature.^54^

### Electrochemistry

Electrochemical measurements were performed using a CHI 1030 potentiostat (CH Instruments, Austin, TX) in a three-electrode arrangement: a glassy carbon electrode (GCE, BASi, Ø = 3 mm) as the working electrode, a Ag/AgCl, 3 M KCl electrode (MINERAL) as the reference (electrolytic bridge filled with 100 mM KNO_3_), and a platinum wire as the counter electrode (MINERAL). The working electrode was sequentially polished with 1.0 and 0.3 μm alumina powder on a polishing cloth to the mirror-like surface, followed by ultrasonication for 1 min in deionized water. All electrochemical measurements were carried out in 100 mM KNO_3_ at room temperature under an argon atmosphere. The samples were prepared separately for each His-Leu:Cu(NO_3_)_2_ ratio. The peptide concentration varied from 0.5 to 2.5 mM, whereas the Cu(NO_3_)_2_ concentration was 0.45 mM for all measurements. The pH was adjusted by adding small amounts of a concentrated KOH or HNO_3_ solution. The applied techniques were CV and DPV. During CV measurements, a scan rate (*v*) of 100 mV/s was applied, whereas the following parameters were used in DPV: pulse amplitude of 0.05 V, pulse width of 0.1 s, sampling width of 0.005 s, and pulse period of 1 s.

### Monitoring the UV–Vis Spectra during the Incubation of Cu(II)/His-Leu Complexes with Ascorbate

The concentrated ascorbate solution in 50 mM HEPES (pH 7.4) was added to solutions containing 0.5 mM CuCl_2_ with 0.6 mM His-Leu or 0.5 mM CuCl_2_ with 2.5 mM His-Leu in 50 mM HEPES (pH 7.4), reaching a final ascorbate concentration of 1 or 5 mM. The UV–vis spectra in the range of 250–900 nm were registered over 24 h, usually every 2 min during the first two hours, every 10 min between the second and fourth hour, and then every 30 min for the final 20 hours. The pH did not change by more than 0.15 pH unit during the incubation, and the experiments were performed under an ambient atmosphere. After the incubation, the selected samples were acidified with formic acid to pH <3 and analyzed using ESI-MS (Q-TOF Premier). The incubation of 0.5 mM CuCl_2_ and 0.6 mM His-Leu with 1 mM ascorbate was also performed in 50 mM phosphate buffer (pH 7.4).

### Ascorbate Oxidation Assay

Oxidation of ascorbate was monitored at 265 nm (*A*_265_) on a Varian Cary 50 spectrophotometer (Agilent) at room temperature and in an ambient atmosphere. First, the baseline signal of 990 μL of 50 mM HEPES (pH 7.4) was recorded at 0, 1, and 2 min. Next, 10 μL of 10 mM AscH^–^ in the same buffer was added, and *A*_265_ monitored for a further 10 min at 1 min intervals, followed by the addition of 5 μL stock solutions of CuCl_2_ or its His-Leu complexes. Then, the *A*_265_ signal was registered every 0.1 min for 60 min. The final concentrations of reagents in the cuvette were 100 μM AscH^–^, 5 μM CuCl_2_, 6, 12.5, 25, 50, and 100 μM His-Leu, and 50 mM HEPES (pH 7.4). The pH of the buffer was checked before the measurements and, if needed, adjusted to 7.4 and verified after the reaction. The initial rate of ascorbate oxidation was calculated on the basis of the slope of the linear fit of the data between the 13th and 14th min of the measurement (thus, between the first and second minute of the oxidation reaction) and the extinction coefficient of ascorbate (ε_265_ = 14 500 M^–1^ cm^–1^).^55^

## Results and Discussion

### Coordination of Cu(II)/His-Leu Complexes

**Figure 1:**
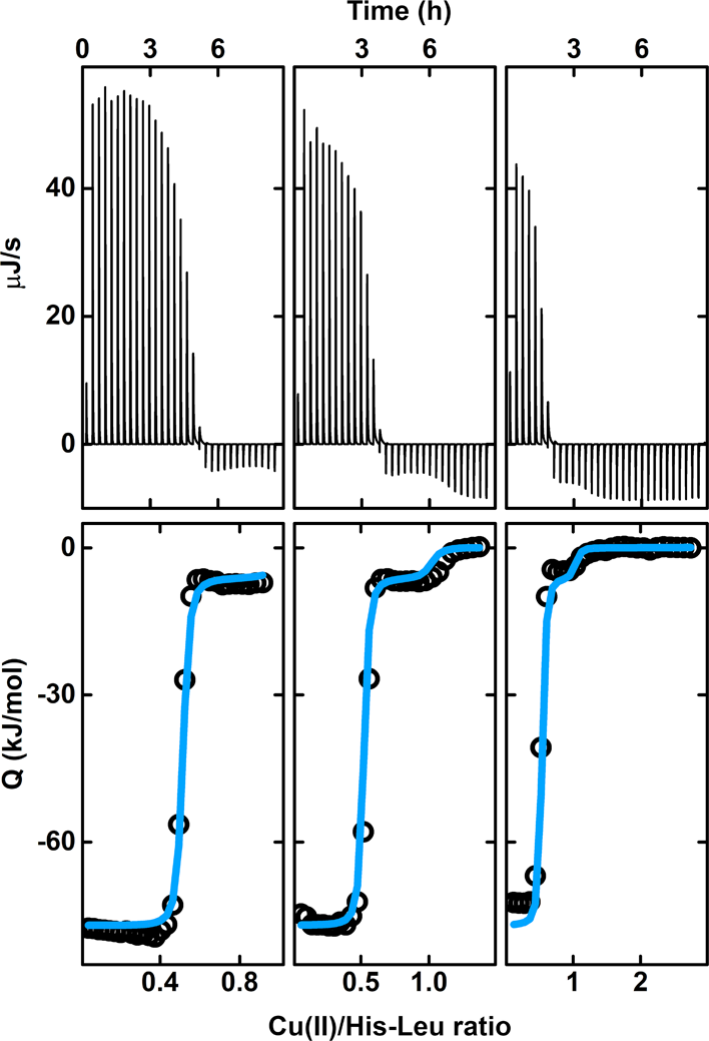
ITC titrations of His-Leu with CuCl_2_ in 20 mM HEPES and 100 mM KNO_3_ at pH 7.4 and 25 °C. The volume of each injection was 16 μL, with the Cu(II) concentration in the syringe being 6 mM. The initial concentrations of His-Leu in the cell were 1.8, 1.2, and 0.6 mM from left to right, respectively. The top plots show the raw experimental data. The bottom plots show the heat in each injection (empty dots), with the global fitting of the model assuming the presence of Cu(His-Leu) and Cu(His-Leu)_2_ complexes (blue lines). Note that the ITC-determined species comprise all protonation states under the given conditions.

We started the study with a series of microcalorimetric titrations, in which a CuCl_2_ solution was added to His-Leu at pH 7.4 (Figure 1). We observed two inflection points at Cu(II):His-Leu ratios of 0.5 and 1.0 in the thermograms. This suggested the formation of complexes with two stoichiometries, namely, Cu(His-Leu)_2_ and Cu(His-Leu). Thermodynamic parameters calculated for the formation of the 1:1 complex were as follows: dissociation constant (*K*_d_), 4.2 ± 1.0 nM; enthalpy change (Δ*H*), −41.6 ± 0.1 kJ/mol. The binding of another His-Leu molecule to the existing 1:1 complex was characterized by a *K*_d_ of 3.2 ± 1.7 μM and a Δ*H* of −35.4 ± 0.1 kJ/mol. The results are presented as the parameter value ± standard deviation from all three experiments. It is noteworthy that the error estimates for *K*_d_ values are probably underestimated, as the used Cu(II)/His-Leu concentrations were not optimal for determining the nanomolar dissociation constant. Moreover, the apparent values may differ slightly from those given here because of the competition for Cu(II) ions with the buffer as well as the potential formation of the ternary Cu(II) complexes between the peptide and HEPES.

**Figure 2:**
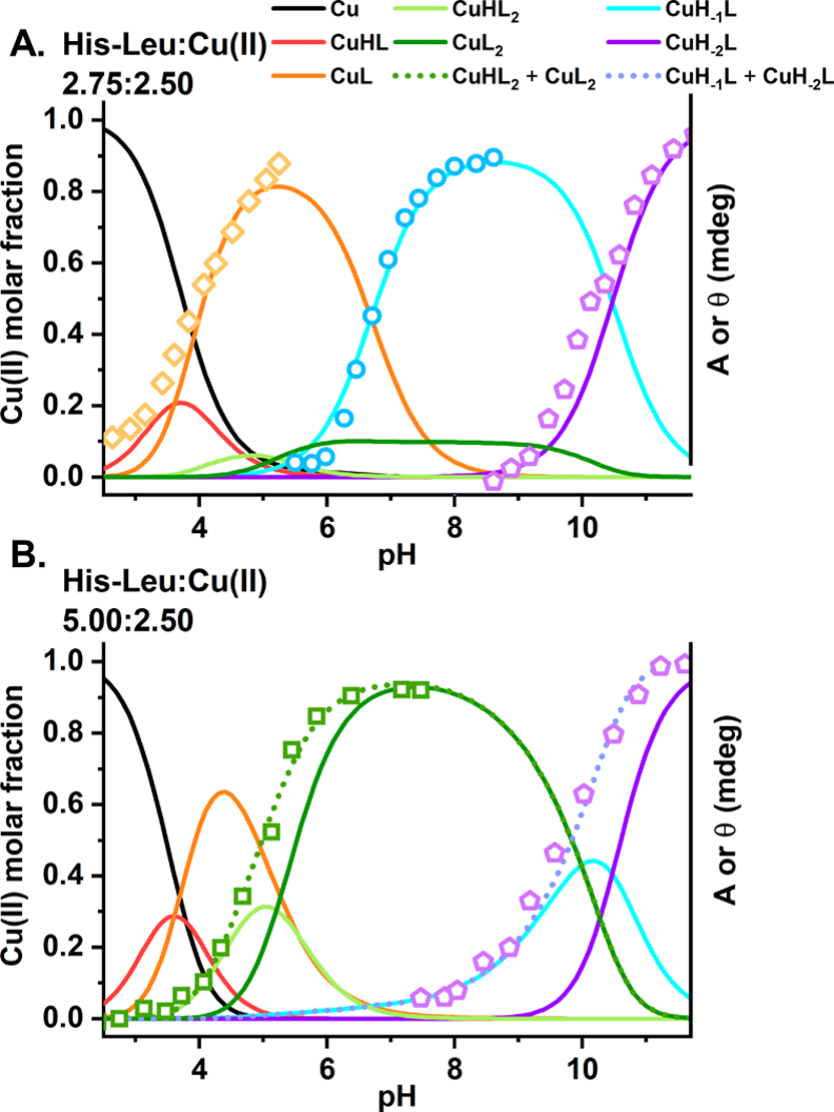
Cu(II) species distributions for Cu(II) complexes of His-Leu calculated for concentrations used in spectroscopic titrations, (A) 2.75 mM His-Leu with 2.50 mM Cu(II) and (B) 5.00 mM His-Leu with 2.50 mM Cu(II), based on stability constants from Table 1. The common scale left-side axes represent the Cu(II) molar fractions. Cu(II) species are color-coded, as described in the figure. The right-side axes provide absorbance and ellipticity obtained in spectroscopic experiments: orange diamonds, A_650_; green squares, θ_700_; blue circles, θ_330_; violet pentagons, θ_500_.

**Figure 3:**
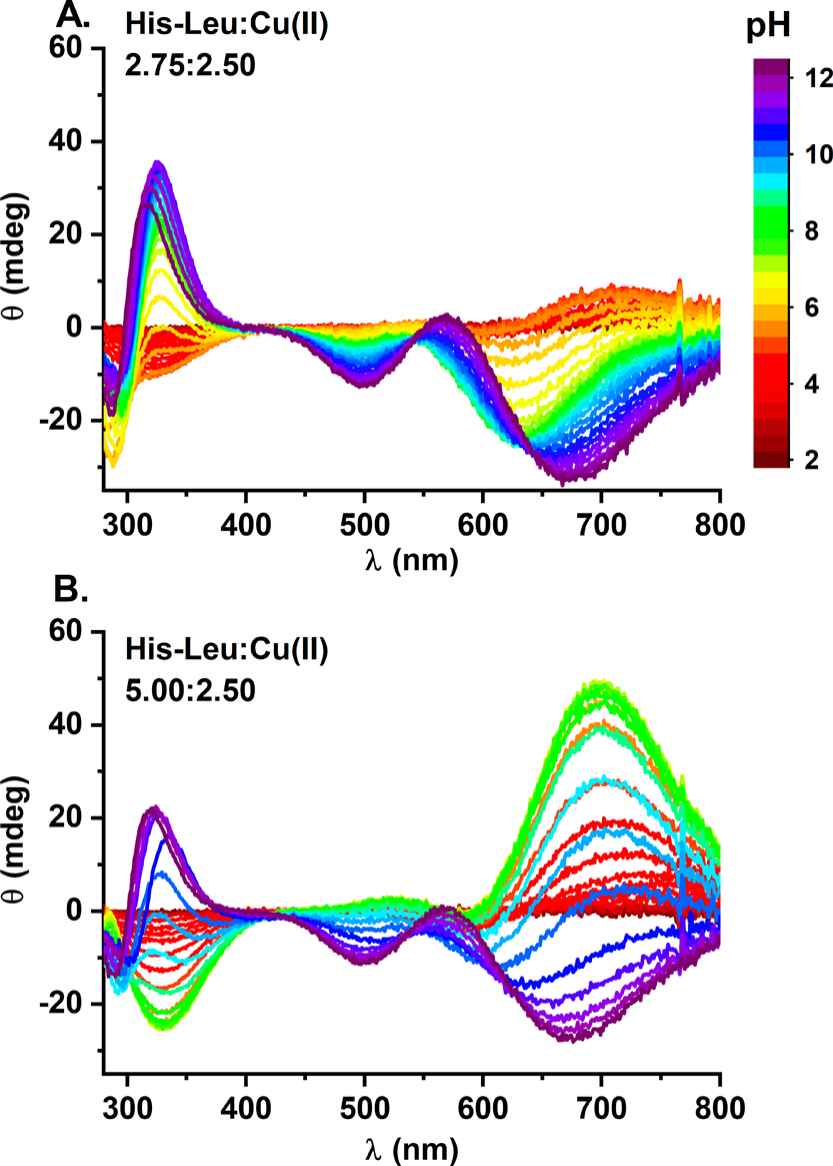
CD titrations of (A) 2.75 mM His-Leu with 2.50 mM Cu(II) and (B) 5.00 mM His-Leu with 2.50 mM Cu(II) with NaOH coded with rainbow colors from red (pH 2) to violet (pH 12) as provided in the figure legend.

Therefore, to delve more deeply into the coordination of Cu(II)/His-Leu complexes, we performed potentiometric and spectroscopic titrations to describe the pH dependence of the binding of Cu(II) to His-Leu and assess the thermodynamic stability of the resulting complexes. Table 1 provides the His-Leu protonation constants and stability constants of the Cu(II) complexes. Figure 2 presents Cu(II) species distributions over the pH range of 2.7–11.5 for two sets of His-Leu and Cu(II) concentrations, 2.75 and 2.50 mM and 5.00 and 2.50 mM, which were used in UV–vis and CD spectroscopic experiments, respectively. The pH-related changes in the CD and UV–vis spectra are shown in Figure 3 and Figure S1, respectively. As the spectra of the Cu(II)/His-Leu complexes varied significantly during the titrations, we also divided spectroscopic results into three pH subranges for easier inspection. They are presented in Figures S2 and S3. In addition, we compared the signals at the two reagent ratios at selected pH values around 4.1, 7.4, and 11.5 (Figure S4).

**Table 1:** Protonation and Stability Constants (log β) for His-Leu (L) and Its Cu(II) Complexes at an Ionic Strength of 0.1 M (KNO_3_) at 25 °C^a^

species	log β	p*K*	assignment of the deprotonation event
HL	7.55(1)	7.55	N^am^ His
H_2_L	13.56(1)	6.01	N^im^ His
H_3_L	16.05(1)	2.49	COO^–^ Leu
CuHL	12.31(2)		
CuL	8.70(1)	3.61	N^am^ His/N^im^ His/COO^–^ Leu
CuH_–1_L	2.00(1)	6.70	N^–^ Leu
CuH_–2_L	–8.47(1)	10.47	OH^–^
CuHL_2_	20.04(3)		
CuL_2_	14.84(1)	5.20	N^im^ His

a Standard deviations in parentheses.

The His-Leu peptide is a H_3_L acid comprising the Leu carboxylic group, the His imidazole ring, and the His amino group. Its protonation constants, listed in Table 1, are consistent with the literature reporting the acid–base properties of His-Xaa dipeptides.^29,30,32,33,35−37,40−45^

**Figure 4:**
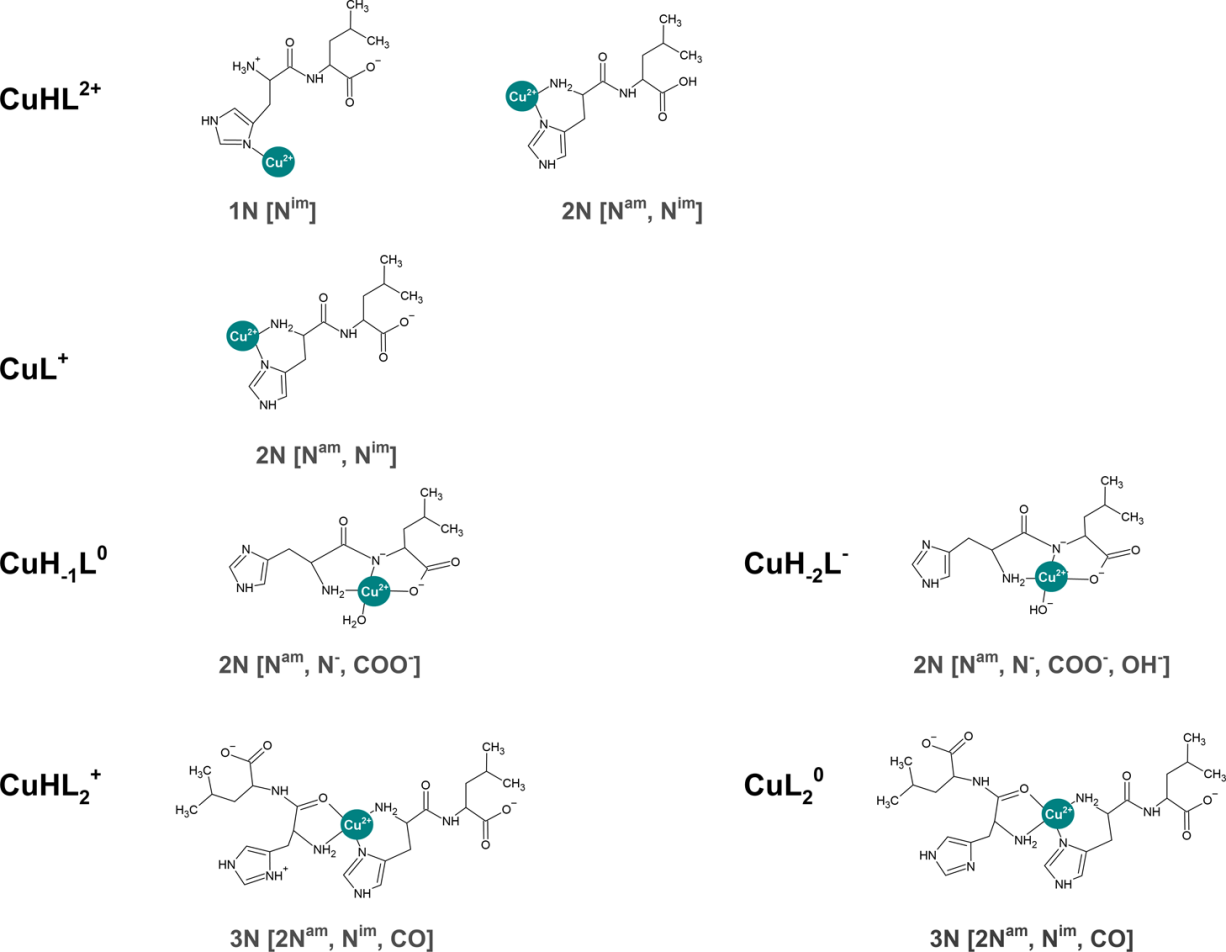
Coordination modes proposed for the Cu(II) complexes of His-Leu. For the sake of clarity, the Cu(II) coordination in the equatorial plane is presented together with water molecules only if their number in the equatorial plane is not higher than one.

The acidic Cu(II)/His-Leu complexes are already present below pH 3. The analysis of potentiometric titrations at low pH indicates the CuHL and CuL stoichiometries, Figure 2. The CuHL species could represent several coordination modes, in which only one nitrogen donor, likely from the imidazole, is engaged in Cu(II) binding. The participation in the coordination of the deprotonated carboxyl oxygen was also suggested in the literature for similar His-Xaa complexes.^33,37,43^ The 2N complex with histamine-like Cu(II) coordination and a protonated Leu carboxyl group is an alternative option. Verification of the CuHL structure(s) on the basis of UV–vis and CD data was problematic due to its small population and the significant overlapping with other species (Figure 2), especially considering the possible mixture of different complexes that could be assigned to CuHL (see the structures proposed in Figure 4).

CuL is the main stoichiometric species at pH 4–6 for a His-Leu:Cu(II) molar ratio of 2.75:2.50 and pH 4–5 for a molar ratio of 5.00:2.50, engaging at its peak >80% of Cu(II) ions around pH 5.3 and >60% at pH 4.4, respectively. Its formation is associated with the blue-shift of the d–d band in UV–vis spectra to ∼670 nm (Table 2 and Figures S1A and S2A). A negative CD band at 330 nm is consistent with N^im^ coordination. The described spectral pattern is characteristic of histamine-like coordination 2N[N^am^, N^im^].^37,56^

**Table 2:** Spectroscopic Parameters of the Cu(II)/His-Leu Species^a^

	UV–vis		CD		EPR			
species	λ (nm)	ε (M^–1^ cm^–1^)	λ (nm)	Δε (M^–1^ cm^–1^)	*g*_iso_	*A*_Cu_ (mT)	*A*_N_ (mT)	binding mode
CuHL	n.d.^b^		n.d.^b^		2.17	3.8	1.7	N^im^
					2.16	5.9	1.6	N^im^ + N^am^
							0.9	
CuL	673	36	328	–0.14	2.15	5.8	1.3	N^im^ + N^am^
							1.1	
CuH_–1_L	605	90	641	–0.36	2.13	3.5	1.6	N^am^ + N^–^ + COO^–^
			496	–0.05			1.2	
			328	+0.35				
CuH_–2_L	622	87	675	–0.39	2.11	3.8	1.6	N^am^ + N^–^ + COO^–^ + OH^–^
			567	+0.03				
			499	–0.15			1.1	
			319	+0.36				
CuHL_2_ or CuL_2_	644	105	698	+0.64	2.1	7.7	1.8	2N^am^ + N^im^ + CO
			332	–0.32			1.1	
							1.1	

a The UV–vis and CD parameters were calculated from experimental spectra using concentrations of individual species obtained from species distribution simulations based on stability constants listed in Table 1. The EPR parameters were obtained by spectral simulations using the EasySpin toolbox for Matlab R2017a.

b Not determined.

Depending on the His-Leu:Cu(II) molar ratio, the CuL complex is replaced by CuH_–1_L for the almost equimolar concentrations of reagents or by bis-complexes CuHL_2_ and CuL_2_ for the 2-fold excess of the peptide over Cu(II) (Figure 2). This difference could be crucial for the physiological roles of Cu(II)/His-Leu complexes as the CuH_–1_L and CuL_2_ species predominate at pH 7.4. The structures of both complexes are notably different, as demonstrated by UV–vis and CD spectra at pH ∼7.4 (Figures S4 and S5). Whereas in CD CuH_–1_L is characterized mainly by the negative band at ∼640 nm and the positive band at 330 nm, the pattern for CuL_2_ is almost mirrored, with a positive band at ∼700 nm and a negative band at 330 nm (Table 2). Correspondingly, in UV–vis, the d–d band of CuL_2_ is red-shifted by almost 40 nm with regard to that of CuH_–1_L. The analogous gradual shift was observed during the titration of the 0.6 mM His-Leu/0.5 mM Cu(II) solution with His-Leu (Figure S6). The excess of the peptide favors the formation of bis-complexes in which the Cu(II) ion can interact with the His1 amino group and the His1 imidazole of two His-Leu molecules. Apparently, only three of four potential nitrogen donors are coordinated, as the d–d band of this species is at a wavelength (624 nm) much higher than that expected for this type of coordination (570 nm).^57^ The analogous situation was reported for other Cu(II)/His-Xaa peptide complexes.^37,43^ Theoretically, the CuL_2_ stoichiometric species could be a mixture of 4N and 3N coordination forms, with one imidazole ligand swapping with the carbonyl oxygen of the peptide bond. This possibility was indicated previously for the Cu(II)/His-Gly system.^38^ For His-Leu, however, the fact that the spectroscopic parameters of CuL_2_ and the minor CuHL_2_ species are apparently identical (see Figure 2, Figure S5, and Table 2) favors the permanent 3N coordination, considering the fact that only the 3N variant is possible for CuHL_2_, which contains one protonated nitrogen group. The coordination of carbonyl oxygen also explains the absence of a CuH_–1_L_2_ species containing a hydroxyl group. This species would have to be formed if there were a water molecule coordinated in CuL_2_, because of the +2 charge on the Cu(II) atom. Furthermore, the carbonyl coordination stipulates the amine binding, due to the formation of a five-membered chelate ring (see Figure 4). The alternative imidazole nitrogen binding would require a seven- or eight-membered ring, both thermodynamically disfavored. The low p*K* of formation of CuL_2_ from CuHL_2_, 5.2, is in line with the considerations described above and can be assigned to the imidazole nitrogen, which is not binding but remains in the vicinity of the Cu(II) ion (Table 1).

The CuL_2_ complex, which cannot be deprotonated without breaking a strong chelate ring, is superseded at high pH by deprotonated mono-complexes. The first of these complexes, CuH_–1_L, is the main species at neutral and weakly alkaline pH in the absence of excess ligand. The binding of the deprotonated peptide nitrogen to Cu(II), rather than a hydroxyl group, is certified by the reversal of the sign of main CD bands, due to the alignment of chiral Cα atoms of both His and Leu on one side of coordination plane. In peptides composed of l-amino acids, this results in the negative d–d band^58−60^ and the positive sign of the CT band at ∼330 nm. It is also confirmed by the low p*K* of CuH_–1_L formation, 6.7. For steric reasons, this peptide nitrogen is paired in coordination with the amine, rather than imidazole nitrogen, by virtue of the formation of a five-membered chelate ring (Figure 4). This structure enables the coordination of carboxylate oxygen in the third coordination site. In the mono-coordination mode, the fourth site is occupied by a water molecule.

In general, this water molecule can be replaced by any donor group of another His-Leu molecule, including the Leu carboxylic group, the His amine, or the His imidazole. The last option could lead to the formation of a binuclear species as was described for the His-Lys and His-Gly complexes.^37,38,43^ This option should be expected to produce a distinct UV–vis and CD spectroscopic pattern, qualitatively similar to that of CuH_–1_L, but blue-shifted due to the stronger ligand field effect.^25^ However, in the case of strong interaction, there would be no monomeric CuH_–1_L, and the species assigned as such would actually be Cu_2_H_–2_L_2_. It is very difficult to discern these two species by potentiometry because their ratios of stoichiometric components are identical.

At a high pH (p*K* = 10.47), a CuH_–2_L species was observed regardless of the reagent molar ratio (Figure 1). The excellent agreement of the spectral pattern at pH ∼11.5 presented in Figure S4 proves that the structure of this complex is indeed alike for the two studied conditions. With the already deprotonated amide, the formation of CuH_–2_L species from CuH_–1_L must be associated with the deprotonation of the equatorially coordinated water molecule (see Figure 4), as reported also for the His-Gly complexes.^33,38^ Its relatively high p*K* value is due to the net neutral charge of Cu(II) in CuH_–1_L, neutralized by the peptide nitrogen and carboxylate donors.

We used room-temperature EPR (rt-EPR) experiments to clarify the issues that were not fully solved by electronic spectroscopies. Cu(II)–Cu(II) dimer formation was treated on the assumption that the formation of a dimeric species enabling Cu(II)–Cu(II) spin coupling would lead to a decrease in EPR signal intensity, as indicated in the literature.^38^ The rt-EPR approach was used instead of a more standard frozen solution spectra, because of a recent finding that bis-complexes may be significantly overrepresented in frozen solutions^23,61^ and because of a strong temperature dependence shown for Cu(II) complexes of other His-1 dipeptides.^31^ The dependence of the rt-EPR spectral intensity at pH 7.4 and a Cu(II):His-Gly ratio near 1, both optimal for putative Cu(II)/His-Gly dimerization, are presented in Figure S7. The EPR signal shape remained unaltered in the measured Cu(II) concentration range of 0.5–10 mM. Its amplitude depended linearly on Cu(II) concentration, as evidenced by the very good quality of the linear fit parameter (*R*^2^ = 0.994) and the absence of systematic deviations from linearity. We can therefore conclude that His-Leu did not form Cu(II) dimers in the tested concentration range.

As mentioned above, the dimeric complexes were observed for His-Gly, and also His-Lys, based on direct EPR-based observations.^38,43,44^ It was also inferred for His-Met, His-Phe, and His-Tyr,^41,42^ and postulated by most authors of early His-Gly studies, on the basis of subtle features of absorption spectra.^30,34,62^ Apparently, the bulk of the branched Leu side chain prevented it more efficiently than the longer but linear side chain of Lys. Similarly, no dimers were detected for Cu(II) complexes of a longer peptide, His-Val-Asp-Gly, which contains a bulky valine residue at position 2. Our results also cast doubt on dimer formation in other bulky systems, as listed above.

**Figure 5:**
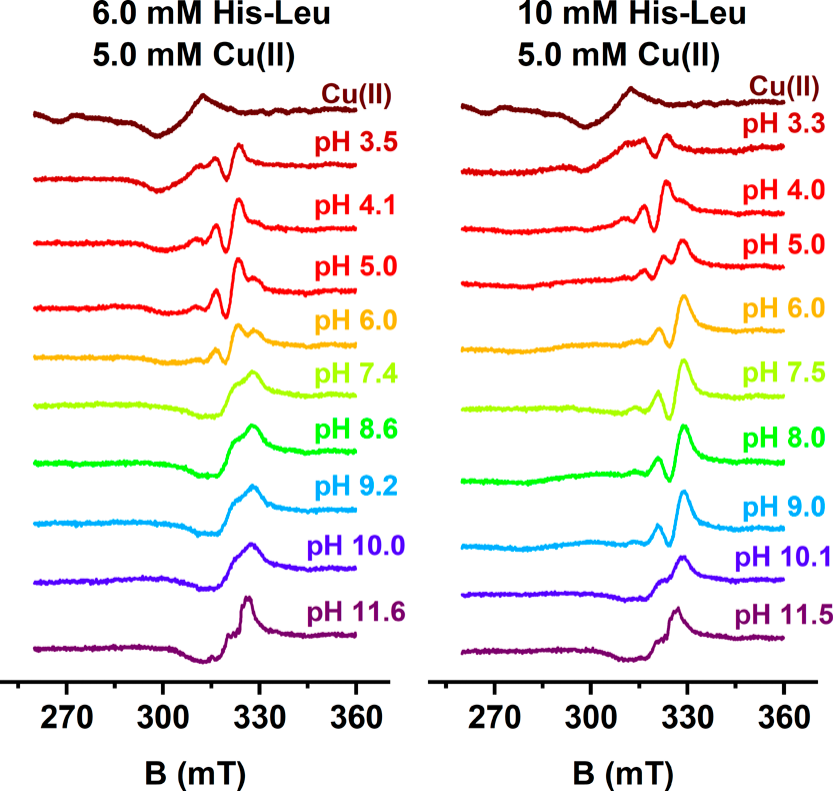
Experimental X-band rt-EPR spectra for 6.0 mM His-Leu with 5.0 mM Cu(II) (left column) and 10 mM His-Leu with 5.0 mM Cu(II) (right column), recorded at 24 *°*C, at pH values indicated in the plot.

The dependence of rt-EPR spectra in a broad pH range at Cu(II):His-Leu ratios of 1:1.2 and 1:2 is presented in Figure 5. The parameters of these spectra derived from simulations are listed in Table 2, along with the UV–vis and CD parameters. The simulated spectra of pure complex species, which are the source of these parameters, are compared to the corresponding experimental spectra in Figure S8. The quality of the derivation was confirmed by the fitted parameters of the Cu(II) aqua ion (*g*_iso_ = 2.21, and *A*_iso_ = 3.86 mT), similar to the published ones.^63^ The parameters of the spectra are in fair agreement with those published previously for the Cu(II)/His-Gly complexes.^31,38^ The analysis of EPR spectra revealed the systematic evolution of the *g*_iso_ and *A*_Cu_ parameters with the strength of the ligand field exerted by nitrogen ligands coordinated to the Cu(II) ion. The assignment of coordination modes was assisted by the A_N_ patterns obtained from spectral simulations, where the highest value of 1.7–1.8 mT could be clearly assigned to the imidazole nitrogen coordination, the lowest value of 0.9–1.1 mT to the amine nitrogen, and the middle value of 1.6 mT to amide nitrogen binding. This observation helped in the proposal of the presence of mixed 1N and 2N coordination modes in the CuHL species, raised above. No indication was found for the non-histamine-like coordination mode in the CuL species. Other complexes identified by electron spectroscopies were confirmed by rt-EPR data, including the identity of coordination modes in CuHL_2_ and CuL_2_ complexes.

The affinity of His-Leu for Cu(II) ions can be described in a simplified way using the conditional binding constant ^C^*K*, valid for a given pH, which allows the comparison of affinity data for complexes with various stoichiometries and obtained by various methodologies.^64,65^ We used the CI approach to calculate the ^C^*K* values using reagent concentrations of 1 and 10 mM from potentiometric data and obtained a log ^C^*K* value of 9.1, corresponding to a *K*_d_ of 0.8 nM (see refs (64) and (65) for the description of the CI approach). These values suggest stronger Cu(II) binding by His-Leu compared to those obtained by ITC (*K*_d_ = 4.2 nM, corresponding to a log ^C^*K* of 8.4). The difference is likely due to the slight interference in the binding of Cu(II) to His-Leu in ITC, exerted by the weakly coordinating HEPES buffer.^66^

### Electrochemical Studies of Cu(II)/His-Leu Systems

**Figure 6:**
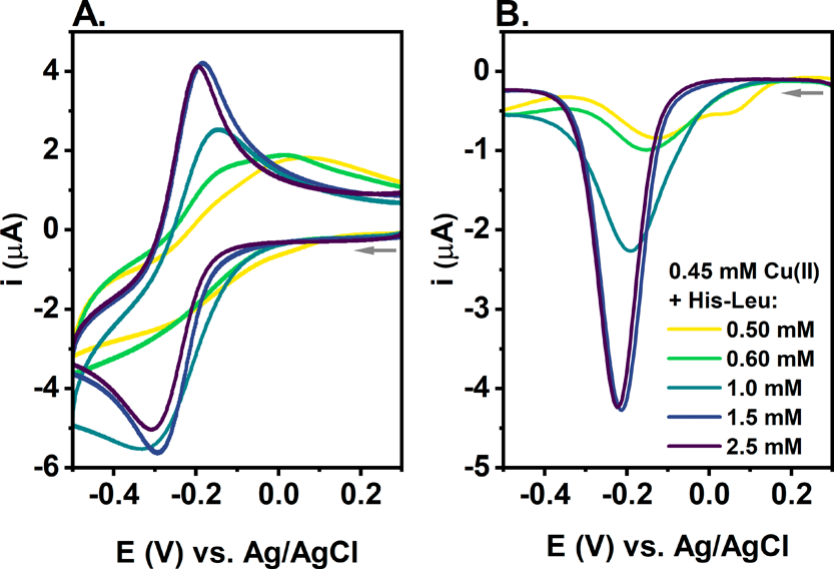
(A) CV and (B) DPV curves of Cu(II) reduction for 0.45 mM Cu(NO_3_)_2_ and 0.50, 0.60, 1.0, 1.5, and 2.5 mM His-Leu in 0.1 M KNO_3_ (pH 7.4). The arrows represent the direction of the potential change.

In the next step, we performed cyclic voltammetry (CV) and differential pulse voltammetry (DPV) measurements at a constant Cu(II) concentration of 0.45 mM, and the His-Leu concentration varied from 0.50 to 2.5 mM. The electrochemical parameters are listed in Tables S1 and S2, while examples of voltammograms are shown in Figure 6 and Figure S9.

Starting from the nearly equimolar system at 0.50 mM His-Leu, we observed the Cu(II) reduction signal at approximately −0.25 V for CV (Figure 6A) and at approximately −0.14 V for DPV (Figure 6B), which could be assigned to CuH_–1_L, the main Cu(II) species under these conditions. The additional DPV signal at approximately 0.06 V (Figure 6B) indicates the presence of other Cu(II) species with higher redox activity, such as CuHL, CuL, or Cu(II) aqua, which is in line with the Cu(II) species distributions calculated for conditions of electrochemical experiments (given in Table S3). This DPV signal disappeared at 0.60 mM His-Leu, likely due to the increasing amount of bis species. Note that Cu(II) ions not bound to the peptide tend to precipitate as Cu(OH)_2_ at pH 7.4^67^ and undergo two-electron reduction to Cu^0^, followed by its deposition at the electrode surface^68^ (see the curves in Figure S10). As higher His-Leu concentrations enabled the dominance of bis species, the Cu(II) reduction potential slightly decreased with a more prominent shift in the Cu(I) oxidation signal, as shown in Figure 6A. As a consequence, the difference between the Cu(II) reduction and Cu(I) oxidation potentials decreased from 0.27 to 0.12 V and the Cu(II) reduction became more reversible (Table S1). Interestingly, among all parameters related to the Cu(II)/Cu(I) cycle, that of Cu(I) oxidation correlates the best with the concentration of the bis species (Figure S11). It could be associated with the potential binding of Cu(I) by His-Leu in a distinct structure, which facilitates the conversion from Cu(I) to the Cu(II) complex at His-Leu ratios of >2:1. For example, the preferential Cu(I) binding by two adjacent His residues (bis-His motif) was previously observed for the truncated Aβ model peptide.^69^ Given the small size of the His-Leu peptide and its high concentrations in electrochemical experiments, we assume that His-Leu could bind Cu(I) ions in a similar manner, engaging His residues from two peptide molecules.

**Figure 7:**
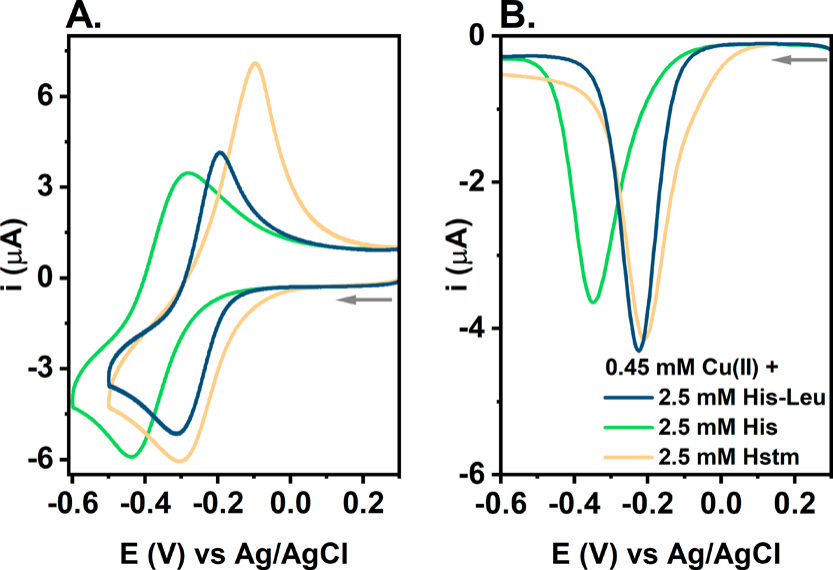
(A) CV and (B) DPV curves of Cu(II) reduction for 0.45 mM Cu(NO_3_)_2_ and 2.5 mM His-Leu (navy), histidine His (green), or histamine Hstm (yellow) in 0.1 M KNO_3_ (pH 7.4). The arrows represent the direction of the potential change.

Then, we compared the CV and DPV curves of Cu(II) complexes of His-Leu with related low-molecular weight substances, histidine and histamine, for an ∼5-fold excess of ligand over Cu(II). The curves are provided in Figure 7. Cu(II) ions are more prone to reduction in the presence of His-Leu than for histidine, with the Cu(II) reduction potential being ∼0.12 V lower for histidine than for His-Leu (Table S1). On the contrary, the Cu(II)/Cu(I) cycle is more reversible for His-Leu (Δ*E* = 0.12 V) than for histamine (Δ*E* = 0.20 V), even though the potential for Cu(II) reduction is similar for both systems, approximately −0.30 V (Figure 7A).

Interestingly, when scanning toward higher potentials of ≤1.2 V, we also noticed an irreversible Cu(II) oxidation signal at ∼0.87 V in DPV curves for the low His-Leu:Cu(II) ratios (Figure S9). Due to its high potential value, we do not expect significant biological consequences of this process. However, from a structural point of view, it aligns with the proposed Cu(II) coordination mode at pH 7.4, which involves the His-Leu amide nitrogen. This signal was barely visible at higher peptide concentrations, which corresponds to the dominance of bis-complexes under this condition (Table S2).

Such electrochemical characteristics of the Cu(II)/His-Leu complexes are distinct from those of Cu(II) complexes of peptides containing the His residue at the second and third positions (His-2 and His-3 peptides, respectively). In the presence of His-2 peptides, the Cu(II) reduction appears at approximately −0.5 V versus Ag/AgCl and is related to major structural changes upon coming back from Cu(I) to Cu(II), as suggested by the large (∼0.5 V) separation of the reduction and oxidation peaks.^61,70,71^ The main species of Cu(II) complexes of His-3 peptides, the 4N complex, is basically redox inert with the Cu(II) reduction occurring below −1.2 V^72^ (and only a very low populated at pH 7.4), but relatively long-lived 2N species was shown to be responsible for the redox activity in this system.^68^ From this perspective, the high redox activity of the Cu(II)/His-Leu system stands out against the properties of Cu(II) complexes of other peptides with His at their N-termini as well as the structurally similar low-molecular weight substances.

### Reactivity of Cu(II)/His-Leu with Ascorbate

**Figure 8:**
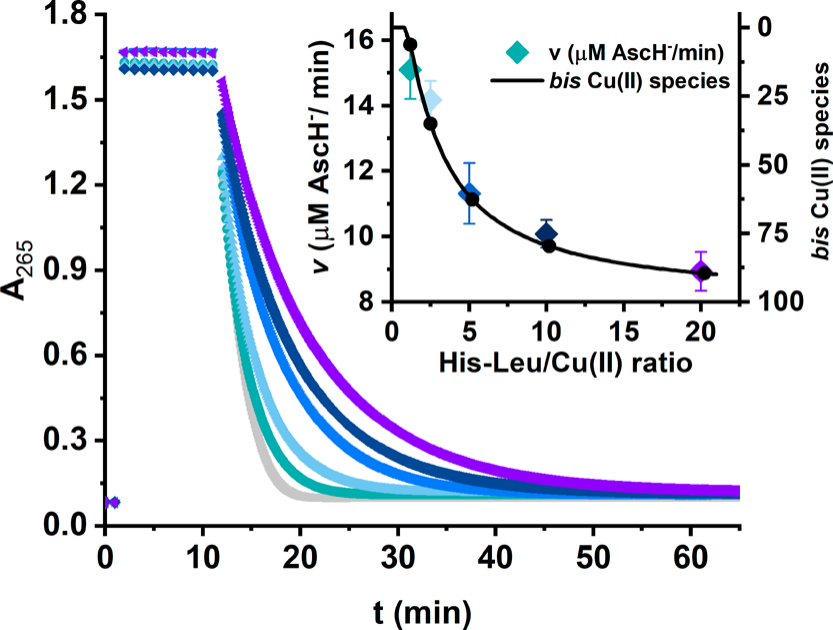
Kinetics of ascorbate oxidation monitored at *A*_265_ in the presence of 5 μM CuCl_2_ (gray points) and the series of Cu/His-Leu solutions (6, 12.5, 25, 50, and 100 μM His-Leu) coded point by point from green to violet. The measurements were performed for 100 μM AscH^–^ in 50 mM HEPES (pH 7.4). The inset represents the initial ascorbate oxidation rate calculated on the basis of the linear fitting of the data between the 13th and 14th minutes of the measurement, overlaid by the molar fraction of bis Cu(II) species (black line) in which the CuL_2_ species predominates [>99% bis Cu(II) species].

Continuing the study of the redox activity of Cu(II) complexes of His-Leu, we investigated the reactivity of those complexes with a physiological reductant, ascorbate. We monitored changes in the d–d band intensity of 0.5 mM Cu(II) with 0.6 mM His-Leu and of 0.5 mM Cu(II) with 2.5 mM His-Leu during their incubation with 1 and 5 mM ascorbate (see the UV–vis spectra in Figure S12). Even a 10-fold molar excess of ascorbate over Cu(II) did not cause the full Cu(II) reduction and disappearance of the d–d band characteristic of Cu(II) complexes. For 0.5 mM Cu(II), 2.5 mM His-Leu, and 1 mM AscH^–^, we noticed an only 12% decrease in the d–d band intensity at the lowest point, whereas the signal decreased by ∼88% for 0.5 mM Cu(II), 0.6 mM His-Leu, and 5 mM AscH^–^ (Figure S13A,C). At the same time, a new band around 400 nm was formed, and its intensity increased over the course of the experiments under all of the conditions studied. A similar band was observed previously during the incubation of Cu(II) complexes of the Ctr1 analogues with ascorbate^73^ or Cu(II) complexes of the histone H2A fragment with H_2_O_2_,^74^ but its identity is still a matter of scientific debate. ESI-MS measurements did not reveal the presence of the oxidized His-Leu peptide, but signals of the oxidized HEPES buffer were noticed (Figure S14). Therefore, we performed analogous measurements in phosphate buffer, where effects of ascorbate on Cu(II)/His-Leu UV–vis spectra were similar to those observed in HEPES (Figure S15). On the contrary, phosphates likely form ternary complexes with mono Cu(II)/His-Leu species due to the significant red-shift observed for spectra in this buffer (Figure S15B). Therefore, we choose HEPES for further experiments, even though it could form additional radicals.^75^ Simultaneously, such an intense signal at 400 nm indicates the high redox activity of Cu(II)/His-Leu, especially the formation of reactive oxygen species (ROS). To investigate this, we performed the ascorbate oxidation assay.^72^ As shown in Figure 8, the Cu(II) salt or the pre-prepared Cu(II)/His-Leu solutions at various molar ratios were introduced into the ascorbate solution at the 12th minute of measurements. In the case of 5 μM CuCl_2_ without the peptide, the oxidation of ascorbate was relatively fast, with an initial rate of 18.6 μM AscH^–^/min. When both Cu(II) and His-Leu were added to ascorbate, the reaction rate decreased by ∼18% for the slight excess of His-Leu over Cu(II) and 2.5 times for the 20-fold excess of His-Leu over Cu(II). Thus, the redox activity of the Cu/His-Leu system was very high across all of the studied His-Leu:Cu ratios; the formation of Cu(II)/His-Leu complexes only slightly diminished the level of ascorbate oxidation by copper for such a high concentration of the chelator. It is noteworthy that the changes in the initial rate of the reaction reflect those in the Cu(II) molar fraction of bis species (see the inset of Figure 8); the larger the contribution of bis species, the lower the initial rate of ascorbate oxidation. On the basis of the species distribution data, we calculated the specific activity of bis species (*v* = 8.4 μM AscH^–^/min) and the activity of mono species (*v* = 16.1 μM AscH^–^/min), which is nearly as high as that of the Cu^2+^ ion.

**Figure 9:**
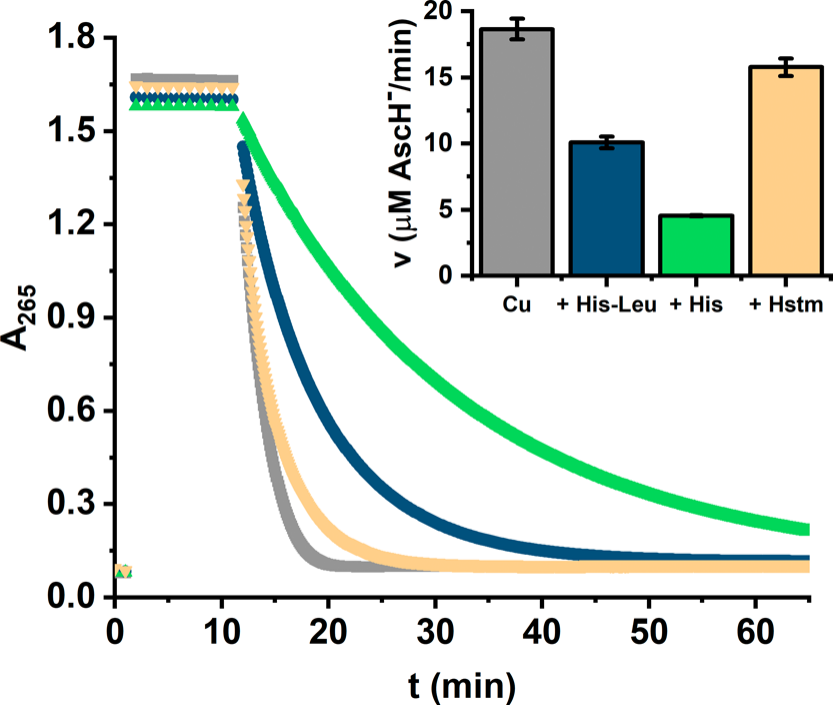
Kinetics of ascorbate oxidation monitored at *A*_265_ in the presence of 5 μM CuCl_2_ alone and in the presence of 50 μM His-Leu, histidine (His), or histamine (Hstm). The measurements were performed for 100 μM AscH^–^ in 50 mM HEPES (pH 7.4). The inset represents the initial ascorbate oxidation rate calculated by linear fitting of the data between the 13th and 14th minutes of the measurement.

Employing a 10-fold molar excess of the ligand over Cu(II), we compared the activity of His-Leu, histidine, and histamine complexes toward ascorbate (Figure 9). The reaction was ∼2 times slower for histidine and ∼50% faster for histamine than for His-Leu. The lower activity in the presence of histidine is in accordance with the lower susceptibility of its complexes to Cu(II) reduction shown in electrochemical experiments (Figure 7). The higher activity in the presence of histamine could not be, however, so straightforwardly explained by a comparison of Cu(II) reduction potentials or the degree of reversibility of the Cu(II)/Cu(I) couple between His-Leu and histamine. As shown in Figure 7 and Table S1, these parameters are very similar for both substances, even suggesting the higher redox activity for His-Leu due to the lower Δ*E* value. However, the ascorbate oxidation assay was conducted at a Cu(II) concentration 100 times lower than those used in electrochemical measurements. As a result, the contribution of Cu(II) mono species increased to >60% for histamine and to ∼20% for His-Leu, whereas it was still <2% for histidine. In addition, there could also be a trace amount of Cu(II) ions not bound to histamine (Table S4). Those facts, together with the generally higher activity of mono species and Cu(II) not bound to the ligands, as described above for His-Leu complexes, are in line with the initial ascorbate oxidation rates presented in Figure 9.

### Biological Relevance

To assess whether His-Leu could have a role as the physiological Cu(II) chelator, we performed a series of theoretical calculations on the basis of the data for Cu(II)/His-Leu complexes obtained by us and literature potentiometric constants for Cu(II) complexes of ligands, which could compete for Cu(II) ions physiologically.

**Figure 10:**
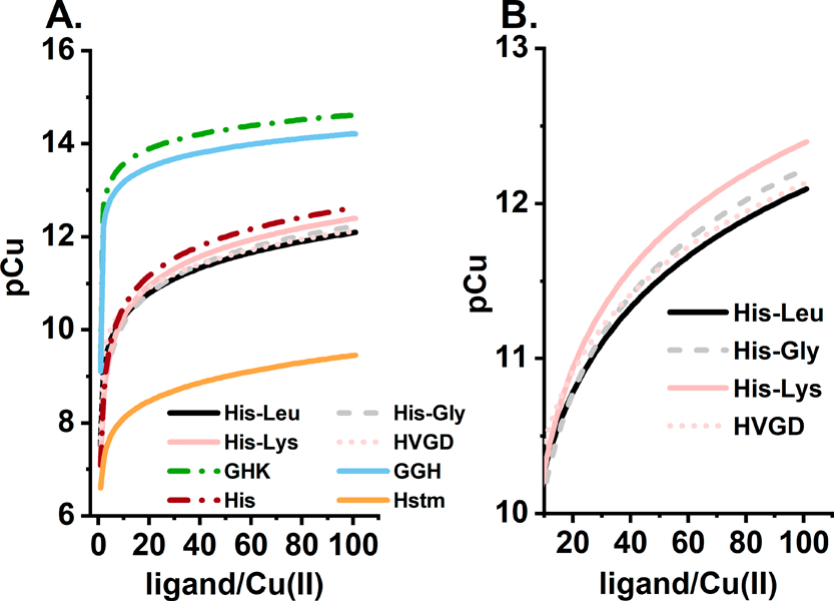
(A) Comparison of the affinity of Cu(II) for selected ligands, given as pCu calculated on the basis of the potentiometric constants [His-Leu, this study; His-Gly,^36^ His-Lys,^43^ HVGD,^76,77^ GHK,^23^ GGH,^19^ histidine (His),^78^ and histamine (Hstm)]^79^ for 1 μM Cu(II) and 1–100 μM ligand at pH 7.4. (B) For the sake of clarity, the curves for His-1 peptides are shown separately for the ligand/Cu(II) molar ratio range of 10–100 μM.

We started with the comparison of their Cu(II) affinity expressed as pCu, which is the negative logarithm of the molar concentration of Cu^2+^ ions at equilibrium with the ligand. In other words, the higher the pCu value, the more stable the Cu(II) complex. This parameter also allows for comparison of the thermodynamic stabilities of Cu(II) complexes of distinct stoichiometries. The analysis of results for 1 μM Cu(II) is given in Figure 10, whereas the analogous comparison for 1 mM Cu(II) is available in Figure S16.

Three distinct ligand subclasses could be distinguished at micromolar Cu(II) concentrations: (i) His-2 and His-3 peptides, (ii) His-1 peptides and histidine, and (iii) histamine. The lower Cu(II) affinity of His-1 peptides, compared to that of the His-2 and His-3 peptides, corresponds to the small amount of bis-complexes for the His-1 peptides under these conditions, which are crucial to enhancing their stability. This is displayed, on the basis of His-Leu, example, in Figure S17. For 1 μM Cu(II) and 10 μM His-Leu, only 44% of Cu(II) ions are engaged in the bis species. In contrast, for 1 mM Cu(II) and 10 mM His-Leu, the fraction of bis species embraces >99% of Cu(II) ions. Consequently, at millimolar concentrations and high ligand:Cu(II) molar ratios, the Cu(II) affinity of His-1 peptides is similar to those of His-2 and His-3 peptides. This analysis supports the statement that the formation of stable bis-complexes is the key factor for His-Leu, along with other His-Xaa dipeptides, to act as relevant Cu(II) ligands.

In the next step, we simulated the competition for Cu(II) between His-Leu and major blood serum Cu(II) binding molecules: human serum albumin (HSA), providing the 4N [N^am^, 2N^–^, N^im^] coordination mode, and representing the high-molecular weight (HMW) Cu(II) pool, and histidine and GHK peptide characteristic for the LMW Cu(II) pool. The calculations were performed for typical concentrations of these ligands: 630 μM HSA,^80^ 600 nM GHK,^81^ and 100 μM His.^17,18^ The Cu(II) concentrations in the HMW and LMW pools (3 μM and 400 nM, respectively) were inferred from a number of studies, as reviewed in ref (28). Published binding constants for HSA,^19^ GHK,^23^ and His^78^ were used along with the His-Leu data obtained here.

**Figure 11:**
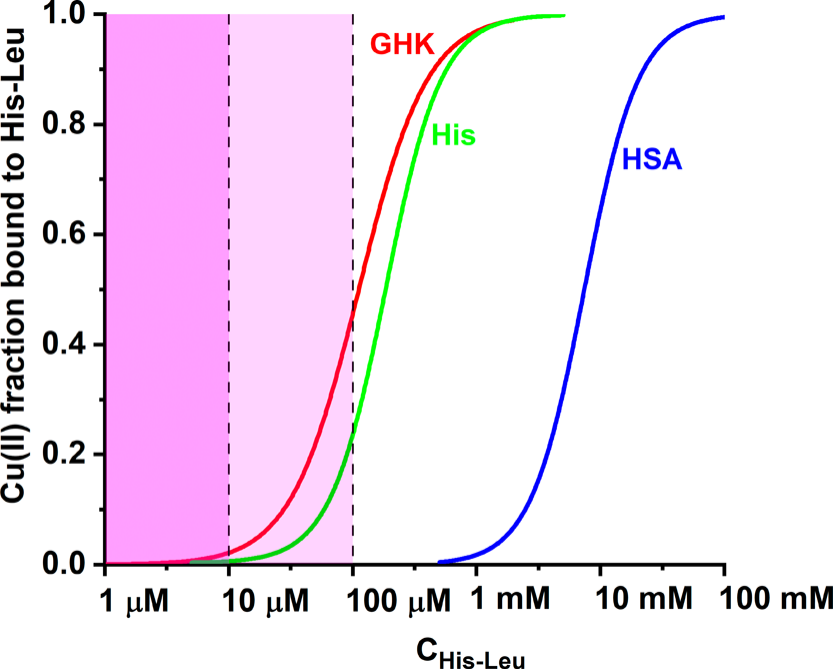
Competition for Cu(II) between His-Leu and major blood serum Cu(II) binding molecules: human serum albumin (HSA), histidine (His), and GHK peptide. The calculations were performed for component concentrations characteristic for high-molecular weight [3 μM Cu(II) and 630 μM HSA] and low-molecular weight [400 nM Cu(II) and 600 nM GHK; 400 nM Cu(II) and 100 μM His] copper pools, using the literature binding constants.^19,23,78^ The shaded field corresponds to the possible His-Leu concentration range in the bloodstream in general (darker) and in kidney circulation (lighter).

The results of these calculations are presented in Figure 11 as competition curves of Cu(II)/His-Leu complexes versus the broad range of His-Leu concentrations. The hypothetical physiological range is marked with a shaded box. In terms of simple competition, HSA-bound Cu(II) ions are beyond the reach of His-Leu, but His-Leu might be a factor in the LMW pool, especially if present in the range of 10–100 μM. Such a His-Leu level is hypothetically possible in the lungs and kidneys^82^ that are the primary sites of its production and also in the course of antihypertension therapies using blockers of angiotensin receptors. Such therapy leads to A2 accumulation^83,84^ and can possibly elevate His-Leu as the accompanying A1 metabolite.^46^ Furthermore, the LMW pool probably comprises ternary complexes,^78^ and His-Leu can be a good candidate for such, on the basis of its readiness to form bis-complexes. Moreover, the kinetic characteristics should also be in favor of His-Leu. As shown for GGH and other simple ATCUN peptides, the Cu(II) binding by 4N structures analogous to those at the HSA N-terminus may take as long as several seconds.^68,85^ The formation of open chelate structures of Cu(II)/His-Leu complexes should be much faster, empowering His-Leu to be a kinetic intermediate in Cu(II) transport. These two issues, ternary complexes and rates of Cu(II) exchange, will be targeted in our future research.

According to the data presented in Figure 9, His-Leu is more redox active than histidine toward ascorbate, and electrochemical data indicate that the His-Leu complex is more prone to reduction than that of His. This feature might make His-Leu a good agent for the delivery of Cu(I) ions to the Ctr1 cellular copper transporter^86^ and/or might contribute to its copper-related toxicity. More work is required to sort out these potential activities.

## Conclusions

His-Leu is a biogenic representative of His-1 peptides formed in the course of angiotensin metabolism. Its concentration in blood remains unknown, but according to physiological data collected in this paper, it may exceed a micromolar level, especially locally in the kidneys and lungs. His-1 peptides are known for their ability to form relatively stable, but redox-capable, Cu(II) complexes; hence, it was interesting to explore a potential of His-Leu to form such complexes and to ascertain their biological relevance. On the basis of a comprehensive set of thermodynamic, calorimetric, spectroscopic, and electrochemical data, we obtained good insight into the stability and redox reactivity of Cu(II)/His-Leu complexes. Simulated competitions for Cu(II) binding between His-Leu and established Cu(II) carriers in blood, HSA, GHK, and histidine indicate that His-Leu may be a part of the LMW Cu(II) pool in blood.

A research issue crucial for the relevance of His-Leu for Cu(II) physiology that emerged from our studies is the formation of ternary complexes with other putative Cu(II) bioligands. Another open question concerns their ability to maintain the Cu(II)/Cu(I) redox couple, which may be biologically deleterious (ROS production) or beneficial (transmembrane copper transport).

## Supporting Information Available

UV–vis titrations of Cu(II)/His-Leu with NaOH, UV–vis and CD spectra at selected pH values, calculated UV–vis, CD, and rt–EPR spectra of Cu(II)/His-Leu species, spectroscopic titrations with His-Leu, dilution rt-EPR experiment, electrochemical curves at 0.3–1.2 V versus Ag/AgCl, electrochemical parameters, Cu(II) species distributions at concentrations of electrochemical and ascorbate oxidation experiments, UV–vis spectra of the incubation of Cu(II)/His-Leu complexes with ascorbate, and comparison of pCu in the milimolar range (PDF)
