# Supplementary material for: The Angiotensin Metabolite His-Leu Is a Strong Copper Chelator Forming Highly Redox Active Species
Source: Inorg Chem. 2024 Jun 15;63(26):12268–80. doi: 10.1021/acs.inorgchem.4c01640 (PMC11220758; doi:10.1021/acs.inorgchem.4c01640)
Supplement: Supplementary file 1 — ic4c01640_si_001.pdf [file ic4c01640_si_001.pdf]

Supporting Information

for

The angiotensin metabolite His-Leu is a strong copper chelator forming highly redox-active species

Nina E. Wezynfeld,[1*] Dobromiła Sudzik,[2] Aleksandra Tobolska,[1] Katerina Makarova,[2,3] Ewelina Stefaniak,[2,4] Tomasz Frączyk,[2] Urszula E. Wawrzyniak,[1] Wojciech Bal[2*]

[1] Chair of Medical Biotechnology, Faculty of Chemistry, Warsaw University of Technology, Noakowskiego 3, 00-664 Warsaw, Poland

[2] Institute of Biochemistry and Biophysics, Polish Academy of Sciences, Pawińskiego 5a, 02-106, Warsaw, Poland

[3] Department of Organic and Physical Chemistry, Faculty of Pharmacy, Medical University of Warsaw, Żwirki i Wigury 61, 02-091 Warsaw, Poland

[4] National Heart and Lung Institute, Imperial College London, Molecular Sciences Research Hub, London, W12 0BZ, United Kingdom

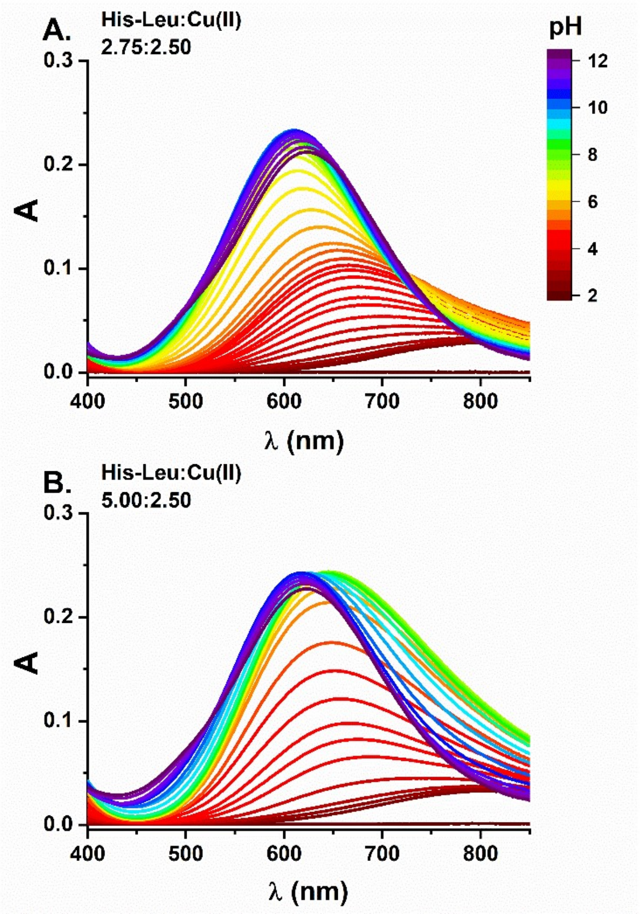

Figure S1. UV−Vis titrations of 2.75 mM His-Leu/2.50 mM Cu(II) (A) and 5.00 mM His-Leu/2.50 mM Cu(II) (B) with NaOH codded with rainbow colours from red (pH 2) to violet (pH 12) as provided in the figure legend.

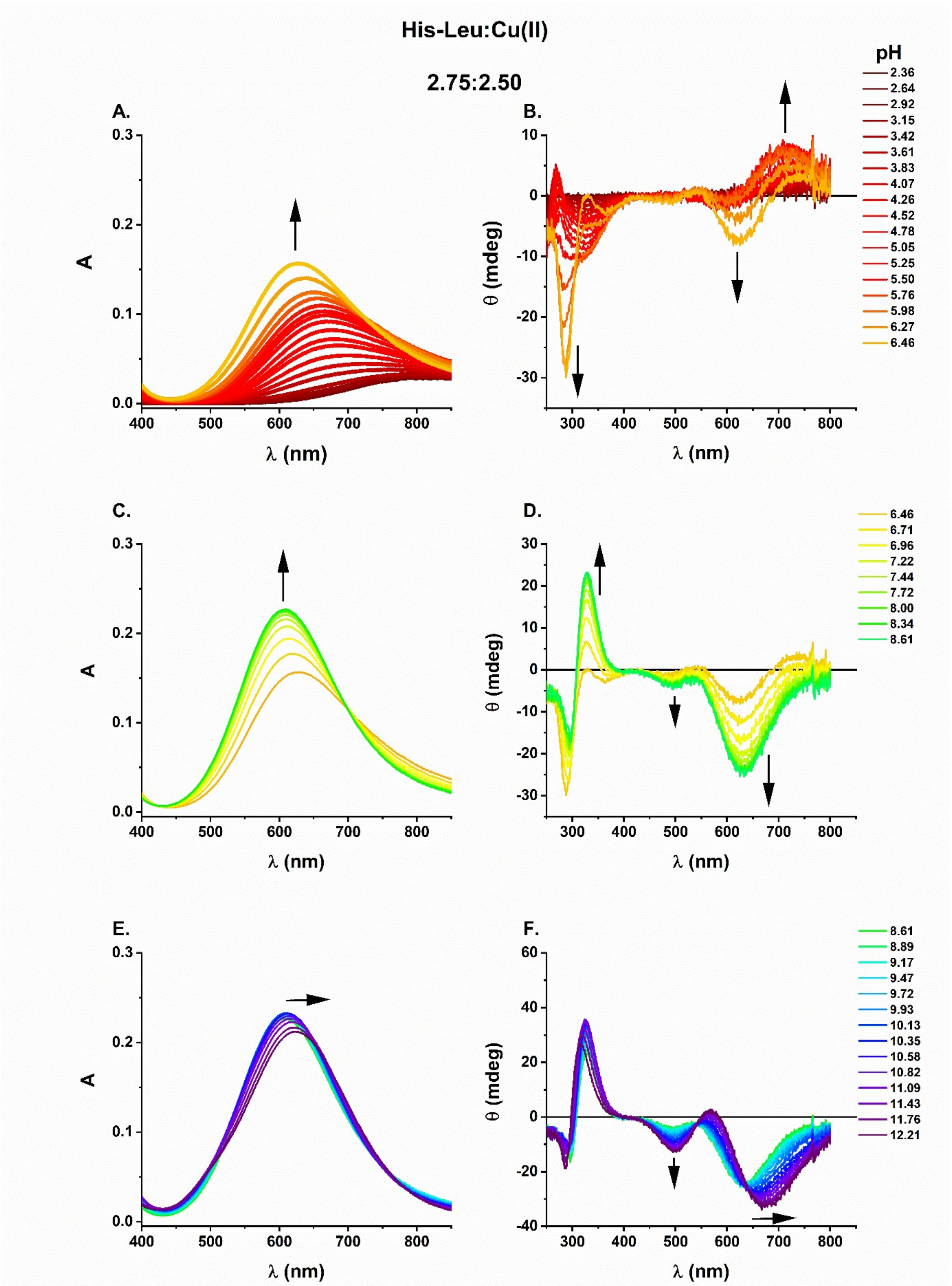

Figure S2. UV−Vis (A, C, E) and CD (B, D, F) spectra of the titrations of 2.75 mM His-Leu/ 2.50 mM Cu(II) with NaOH divided into three pH subranges, pH 2.4−6.5 (A, B), pH 6.5−8.6 (C, D), and pH 8.6−12.2 (E, F) codded with rainbow colours as provided in the figure legends. The arrows represent the direction of spectral changes in the given pH subrange.

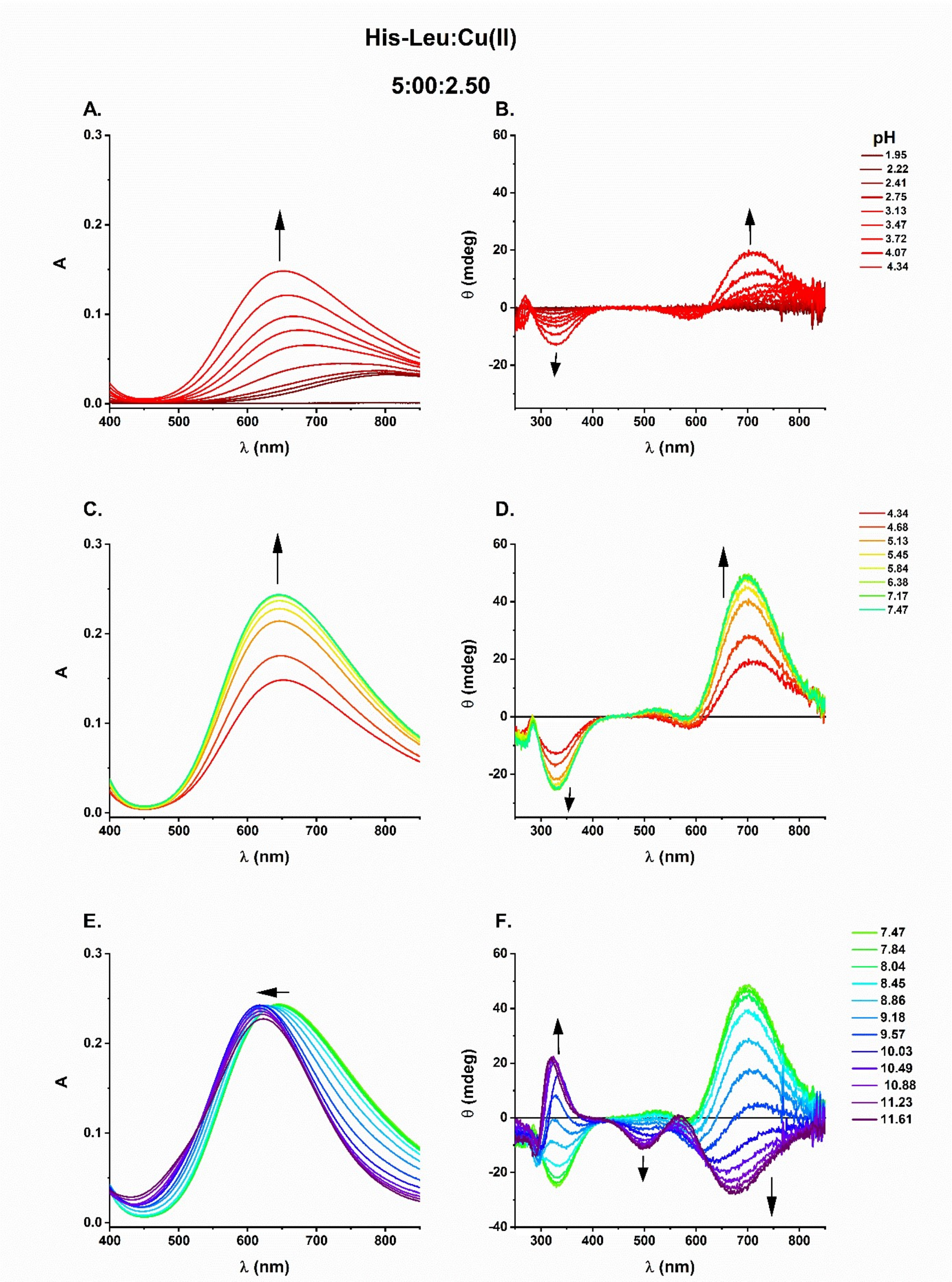

Figure S3. UV−Vis (A, C, E) and CD (B, D, F) spectra of the titrations of 5.00 mM His-Leu/ 2.50 mM Cu(II) with NaOH divided into three pH subranges, pH 2.0−4.3 (A, B), pH 4.3−7.5 (C, D), and pH 7.5−11.6 (E, F) codded with rainbow colours as provided in the figure legends. The arrows represent the direction of spectral changes in the given pH subrange.

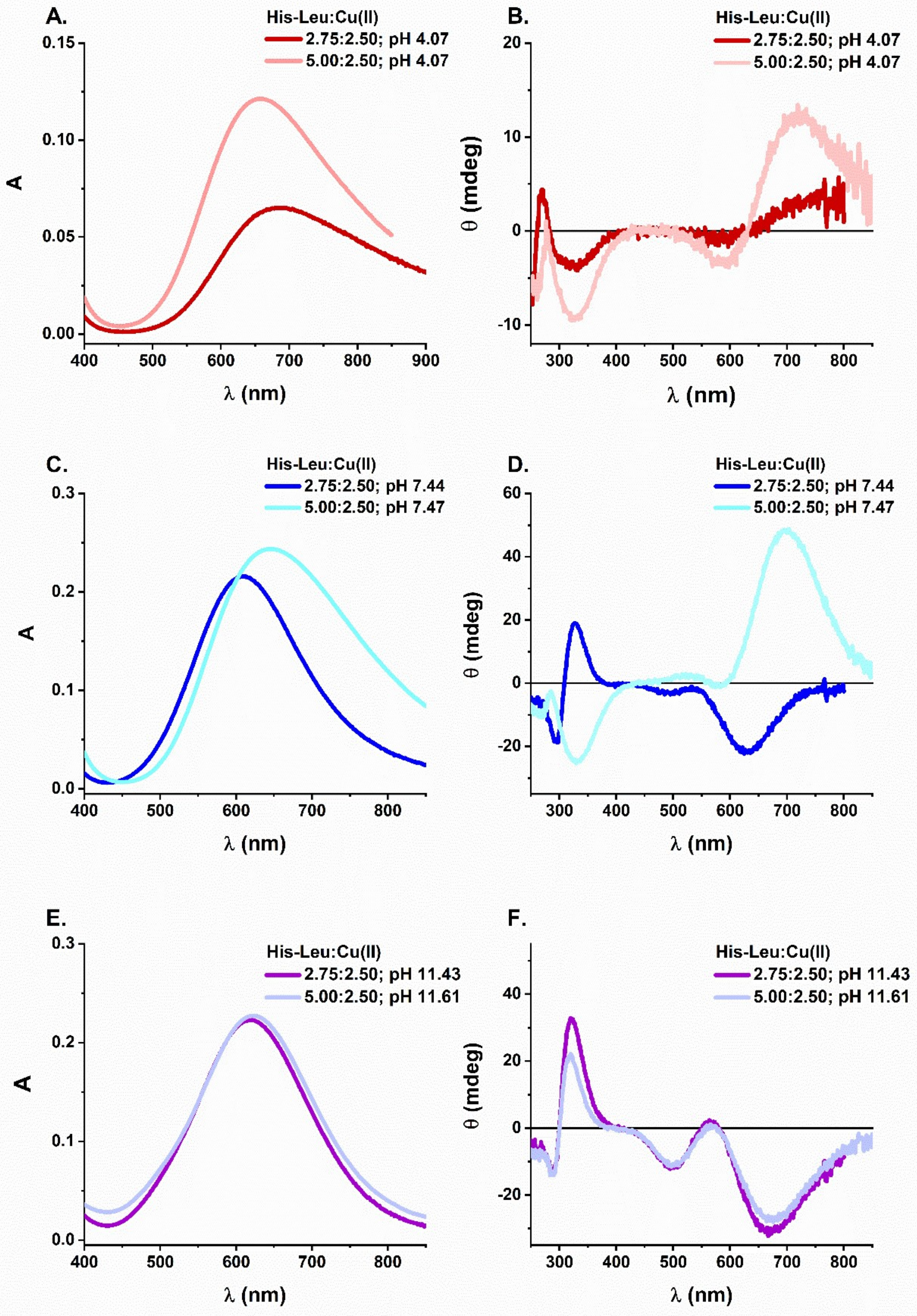

Figure S4. UV−Vis (left) and CD (right) spectra of 2.75 mM His-Leu/2.50 mM Cu(II) (light−coloured curves) and 5.00 mM His-Leu/2.50 mM Cu(II) (dark−coloured curves) at selected pH values around 4.0 (A, B), 7.4 (C, D), and 11.5 (E, F).

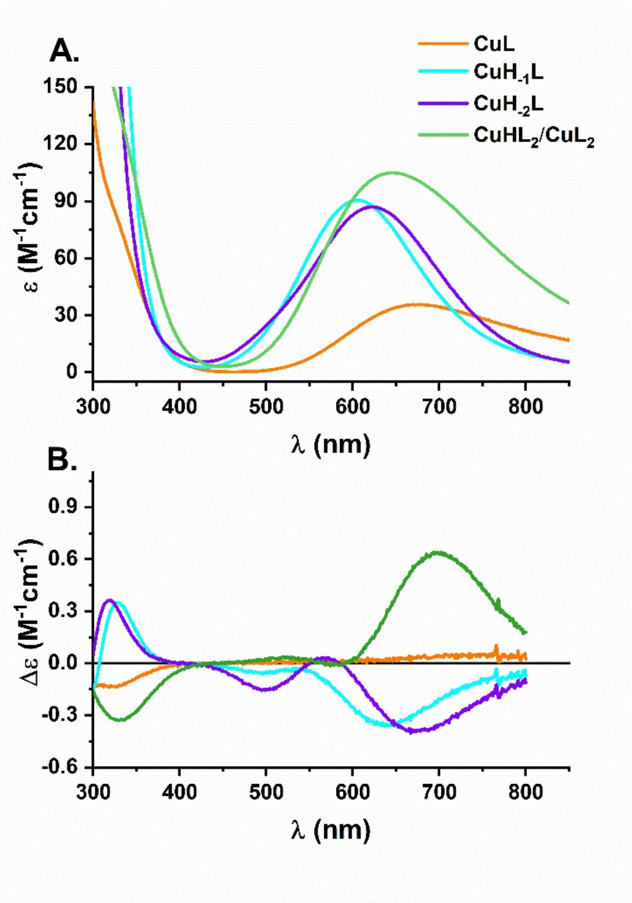

Figure S5. Calculated UV−vis (A) and CD (B) spectra of Cu(II)/His-Leu species.

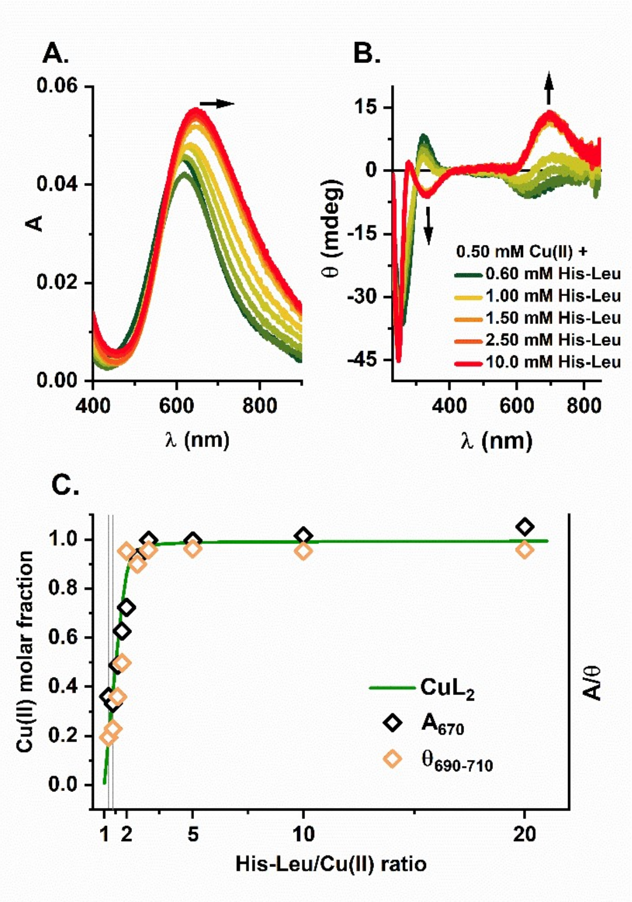

Figure S6. UV−Vis (A) and CD (B) spectra of the titration of 0.6 mM His-Leu/0.5 mM Cu(II) with His-Leu at pH 7.4. The selected final concentrations of the peptide in the cuvette for the given spectrum are provided in the legend. The arrows represent the direction of spectral changes during the experiment. (C) The comparison of CuL_2 molar fraction with the spectral changes (A_{670} and $\theta_{690-710}$) registered during the titration.

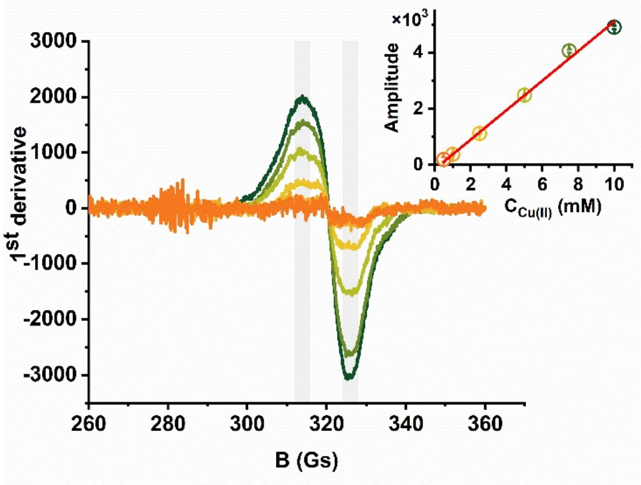

Figure S7. Concentration dependence of rt−EPR spectra recorded at 24 °C for solutions containing Cu(II) and His-Leu at pH 7.4 and the 1.0:1.2 molar ratio, and Cu(II) concentrations of 10.0, 7.5, 5.0, 2.5, 1.0, and 0.5 mM codded with the gradient colours from dark green (10.0 mM Cu(II)) to orange (0.5 mM Cu(II)). Inset shows concentration dependence of signal amplitude, obtained as a difference of integrated 1st derivative amplitudes integrated over the shaded spectral ranges, and its linear fit ($R^2 = 0.994$).

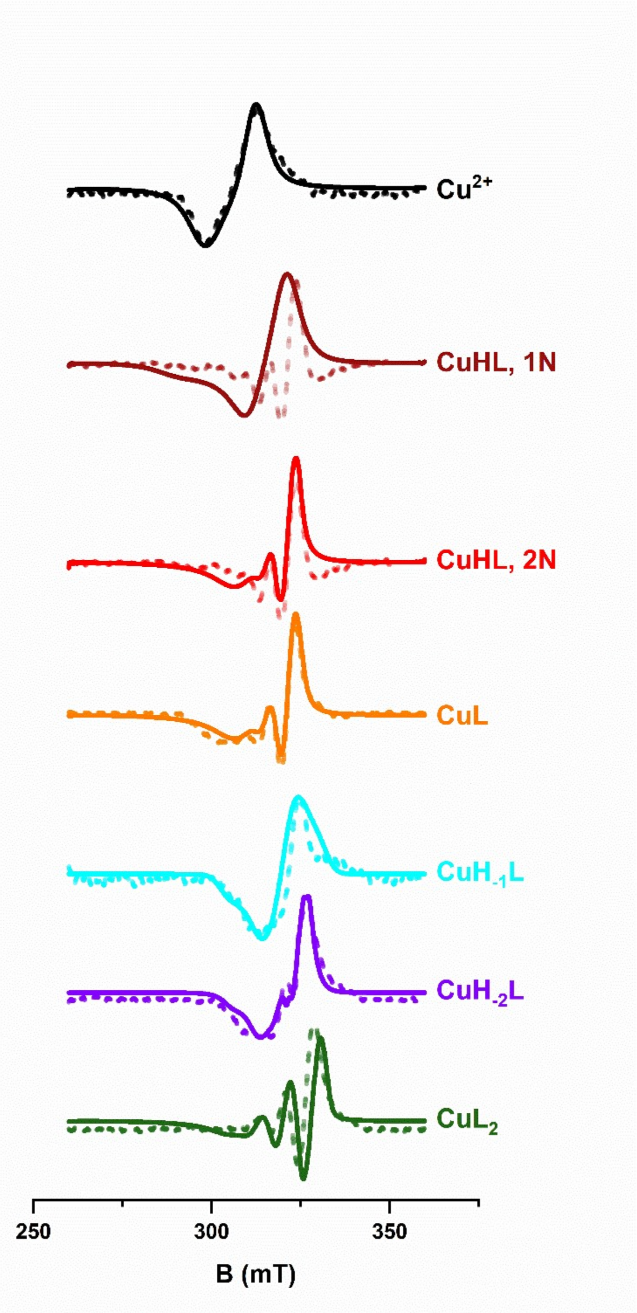

Figure S8. The rt−EPR spectra simulated (solid lines) for individual complex species using EasySpin, compared with their experimental counterparts (dashed lines).

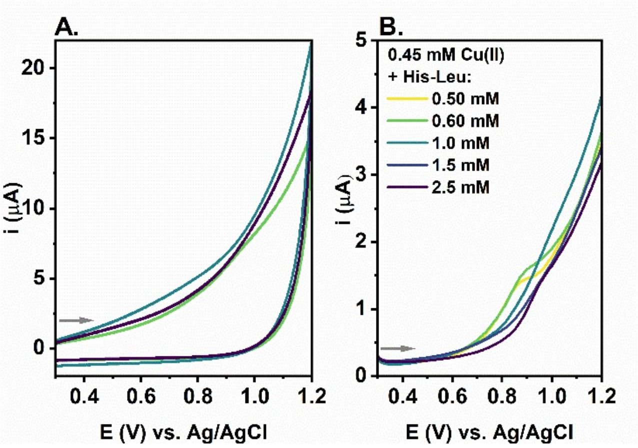

Figure S9. CV (A) and DPV (B) curves of Cu(II) oxidation for 0.45 mM $Cu(NO_3)_2$ and 0.50 mM/0.60 mM/1.0 mM/1.5 mM/2.5 mM His-Leu in 0.1 M KNO_3 pH 7.4. The arrows represent the direction of potential change.

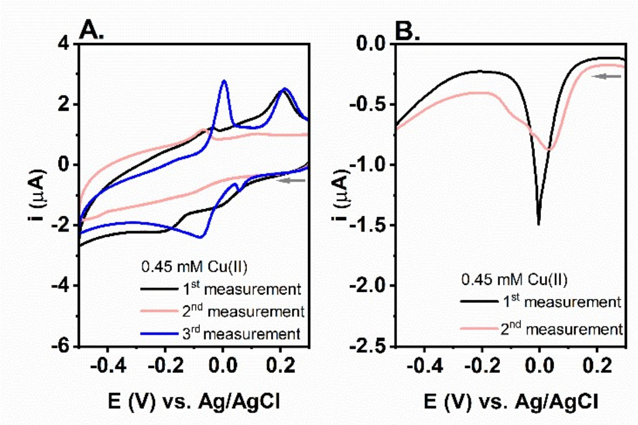

Figure S10. CV (A) and DPV (B) curves registered after the addition of 0.45 mM $Cu(NO_3)_2$ to 0.1 M KNO_3 and pH adjusting to 7.4. The arrows represent the direction of potential change.

Table S1. Potential and current values of redox processes obtained by cyclic voltammetry for 0.45 mM Cu(II) and ligands, His-Leu, histidine (His) and histamine (Hstm), at concentrations given in the Table. The measurements for 0.50 mM His-Leu and 2.5 mM ligands were repeated at least three times and the respective standard deviation values at the last significant digit are in the parenthesis.

ligand	C_{ligand} (mM)	CV						
		$E_{Cu(II)/Cu(I)}$ (V)	$I_{Cu(II)/Cu(I)}$ (μA)	$E_{Cu(I)/Cu(II)}$ (V)	$I_{Cu(I)/Cu(II)}$ (μA)	$\Delta E_{Cu(II)/Cu(I)}$ (V)	$E_{Cu(II)/Cu(III)}$ (V)	$I_{Cu(II)/Cu(III)}$ (μA)
**His-Leu**	**0.50**	**-0.25(1)**	**-0.6(2)**	**0.025(1)**	**1.9(2)**	**0.27(1)**	**0.95(1)**	**3.7(4)**
	0.60	-0.260	-0.96	0.015	1.86	0.275	0.947	4.03
	1.0	-0.324	-4.77	-0.153	3.00	0.171	-	
	1.5	-0.293	-4.96	-0.184	4.16	0.109	-	
	**2.5**	**-0.31(1)**	**-4.6(2)**	**-0.19(1)**	**4.12(1)**	**0.12(1)**	**-**	
**His**	**2.5**	**-0.43(1)**	**-4.5(4)**	**-0.28(1)**	**3.7(3)**	**0.15(1)**	**-**	
**Hstm**	**2.5**	**-0.30(1)**	**-4.2(3)**	**-0.10(1)**	**6.8(8)**	**0.20(1)**	**-**	

Table S2. Potential and current values of redox processes obtained by differential pulse voltammetry for 0.45 mM Cu(II) and ligands, His-Leu, histidine (His) and histamine (Hstm), at concentrations given in the Table. The measurements for 0.50 mM His-Leu and 2.5 mM ligands were repeated at least three times and the respective standard deviation values at the last significant digit are in the parenthesis.

ligand	C_{ligand} (mM)	DPV			
		E_{red} (V)	I_{red} (μA)	E_{ox} (V)	I_{ox} (μA)
**His-Leu**	**0.5**	**-0.14(1)**	**-0.57(1)**	**0.87(1)**	**0.21(1)**
	0.6	-0.148	-0.63	0.880	0.16
	1.0	-0.192	-1.98	-	-
	1.5	-0.212	-4.10	-	-
	**2.5**	**-0.22(1)**	**-4.15(9)**	**0.95(1)***	**0.09(1)***
**His**	**2.5**	**-0.35(1)**	**-3.9(3)**	**-**	**-**
**Hstm**	**2.5**	**-0,21(1)**	**-3,7(6)**	**-**	**-**

*the low intensity signal noticed by DPV and CV measurements at v = 10 mV/s

Table S3. Cu(II) species distributions for Cu(II) complexes of His-Leu calculated for concentrations used in electrochemical measurements and pH 7.4

$C_{Cu(II)}$ (mM)	$C_{His\text{-}Leu}$ (mM)	% Cu(II) molar fraction						
		Cu	CuHL	CuL	$CuH_{-1}L$	$CuH_{-2}L$	$CuHL_2$	CuL_2
0.45	0.50	0.056	0.002	14.8	74.2	0.063	0.068	10.8
0.45	0.60	0.011	0.002	11.3	56.5	0.048	0.202	32.0
0.45	1.0	<0.001	<0.001	1.23	6.18	0.005	0.580	92.0
0.45	1.5	<0.001	<0.001	0.288	1.44	0.001	0.616	97.6
0.45	2.5	<0.001	<0.001	0.110	0.552	0.0005	0.623	98.7

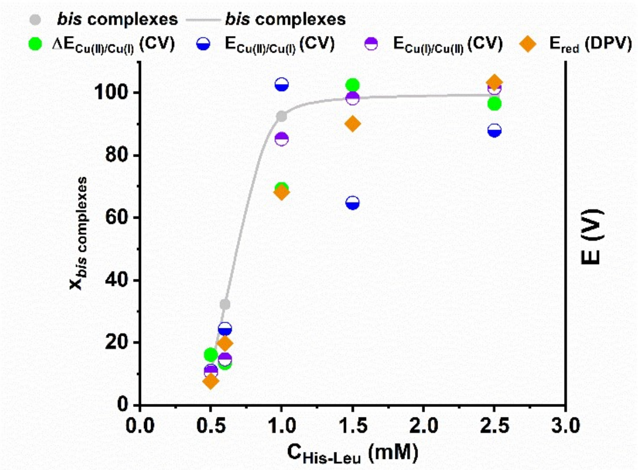

Figure S11. Comparison between the Cu(II) molar fractions of *bis* complexes at conditions of electrochemical measurements and CV (circles) or DPV (diamonds) potential values. The scale for the right Y-axis was omitted as it represents several Y-axis for different redox processes, which cover various ranges of E values.

Table S4. Cu(II) species distributions for Cu(II), His-Leu, histamine (Hstm), histidine (His) concentrations and pH 7.4 used in the ascorbate oxidation assay

**composition Cu(II)/L**	**% Cu(II) molar fraction**		
	**Cu(II) not bound to L**	***mono* species**	***bis* species**
5 μM Cu(II)/ 6 μM His-Leu	0.1%	93.8%	6.1%
5 μM Cu(II)/ 12.5 μM His-Leu	< 0.1%	65.0%	35.0%
5 μM Cu(II)/ 25 μM His-Leu	< 0.1%	38.7%	61.3%
5 μM Cu(II)/ 50 μM His-Leu	< 0.1%	20.7%	79.3%
5 μM Cu(II)/ 100 μM His-Leu	< 0.1%	10.6%	89.3%
5 μM Cu(II)/ 50 μM His[a]	<0.1%	1.7%	98.3%
5 μM Cu(II)/ 50 μM Hstm[b]	0.1%	63.6%	36.3%

[a] calculated based on literature potentiometric results[1]

[b] calculated based on literature potentiometric results[2]

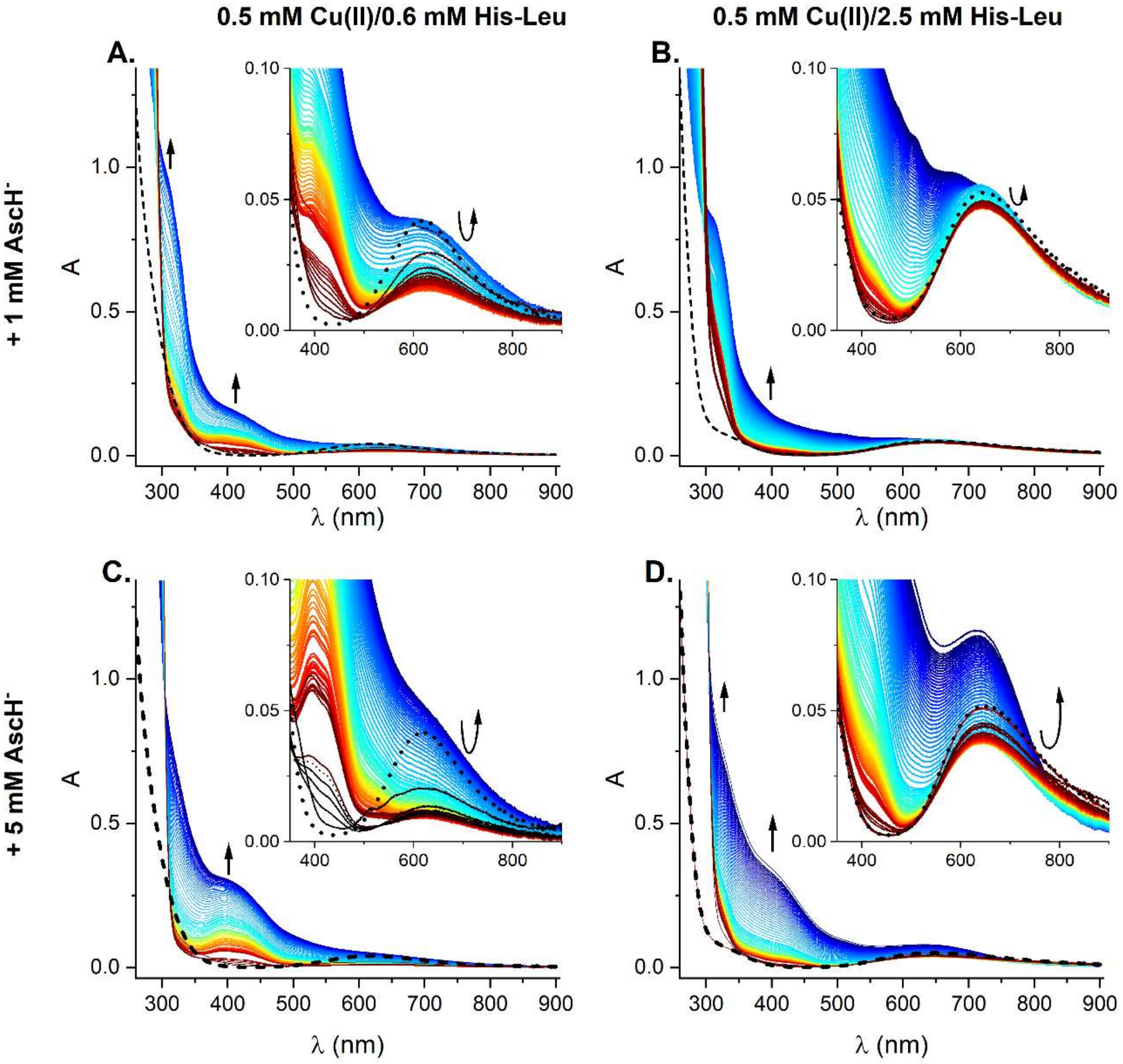

Figure S12. UV-vis spectra recorded during the incubation of Cu(II)/His-Leu with ascorbate at four reagent concentrations, 0.5 mM Cu(II)/0.6 mM His-Leu/1 mM $AscH^-$ (A), 0.5 mM Cu(II)/2.5 mM His-Leu/1 mM $AscH^-$ (B), 0.5 mM Cu(II)/0.6 mM His-Leu/5 mM $AscH^-$ (C), and 0.5 mM Cu(II)/2.5 mM His-Leu/5 mM $AscH^-$ (D). All incubations were performed for 24 hours in 50 mM HEPES pH 7.4. The dashed spectra refer to signals for Cu(II)/His-Leu complexes without $AscH^-$. The spectra after the $AscH^-$ addition are codded with rainbow colors from dark red to blue. The insets are for zooming the changes near the *d-d* region. The arrows indicate the direction of the band changes during the reaction.

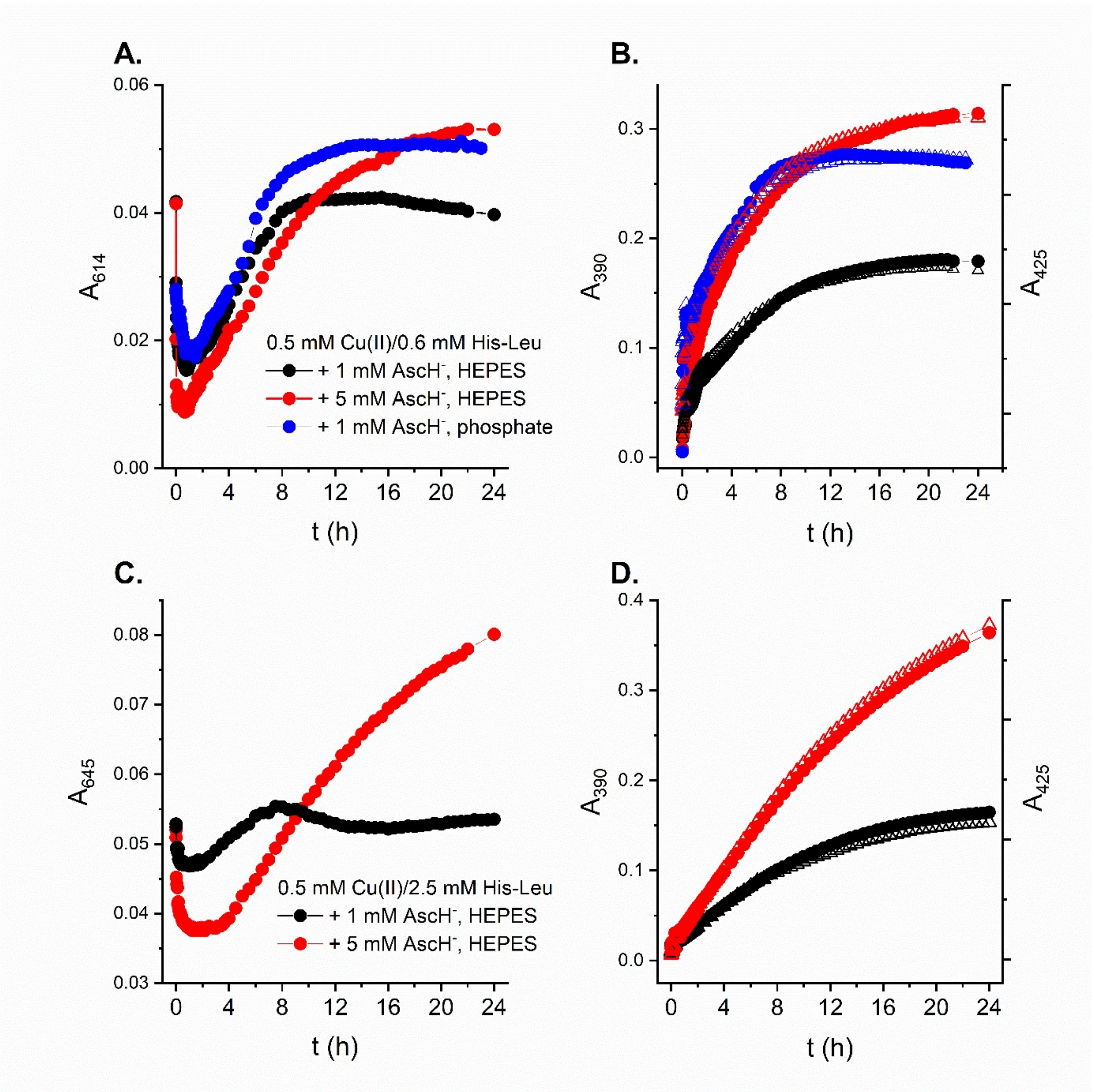

Figure S13. The changes in absorbance for d-d bands (A, C) and for the band at around 400 nm (B, D) during 24-hour kinetics recorded for 0.5 mM Cu(II)/0.6 mM His-Leu (A, B) and 0.5 mM Cu(II)/2.5 mM His-Leu (C, D) after the addition of 1 mM $AscH^-$ (black points for the reaction in 50 mM HEPES pH 7.4 and blue points for the reaction in 50 mM phosphate pH 7.4) or the addition of 5 mM $AscH^-$ (red points for the reaction in 50 mM HEPES pH 7.4). The band around 400 nm comprises two maxima at 390 nm and 425 nm, whose intensity increases proportionally to each other, as shown in B and D, where dot symbols representing absorbance at 390 nm are overlapped by triangles representing absorbance at 425 nm.

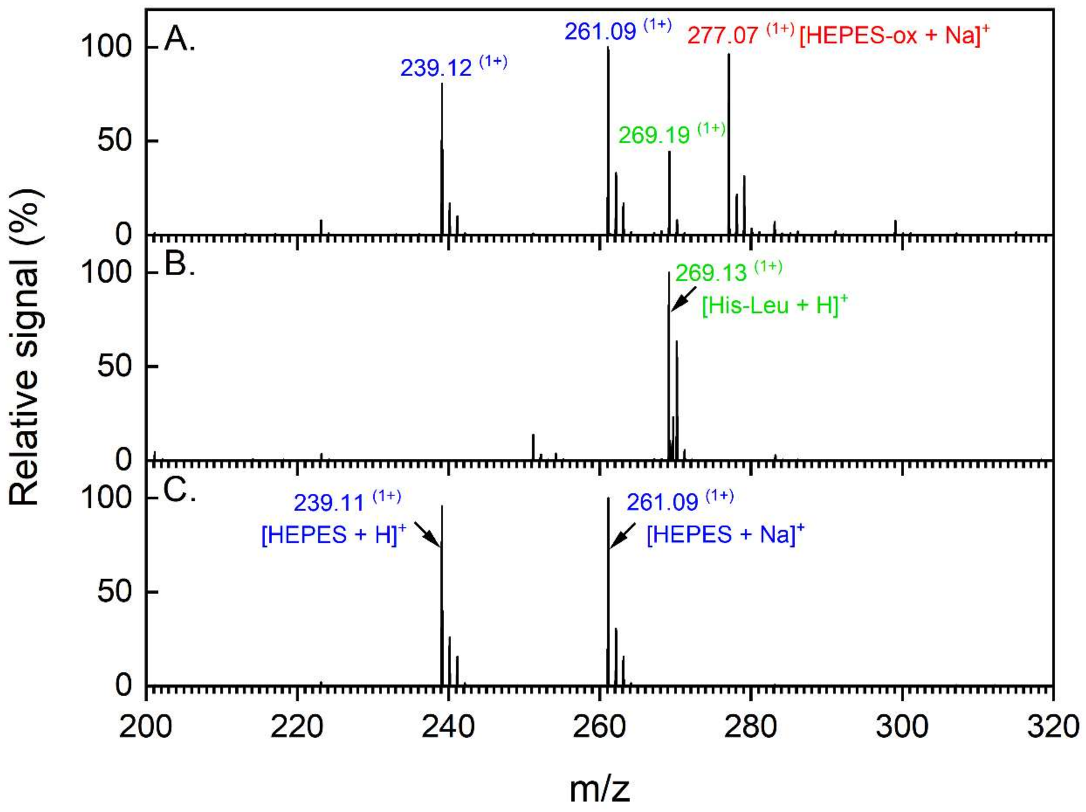

Figure S14. ESI-MS spectra of the solution of 0.5 mM Cu(II), 0.6 mM His-Leu, after the reaction with 1 mM ascorbate in 50 mM HEPES (A), fresh His-Leu (B), and HEPES/Na (C), all acidified with formic acid just before measurement. Detected m/z values are for His-Leu (269.13, 269.19), HEPES (239.11, 239.12), Na-adduct of HEPES (261.09), and probable Na-adduct of oxidized (+16) HEPES (277.07).

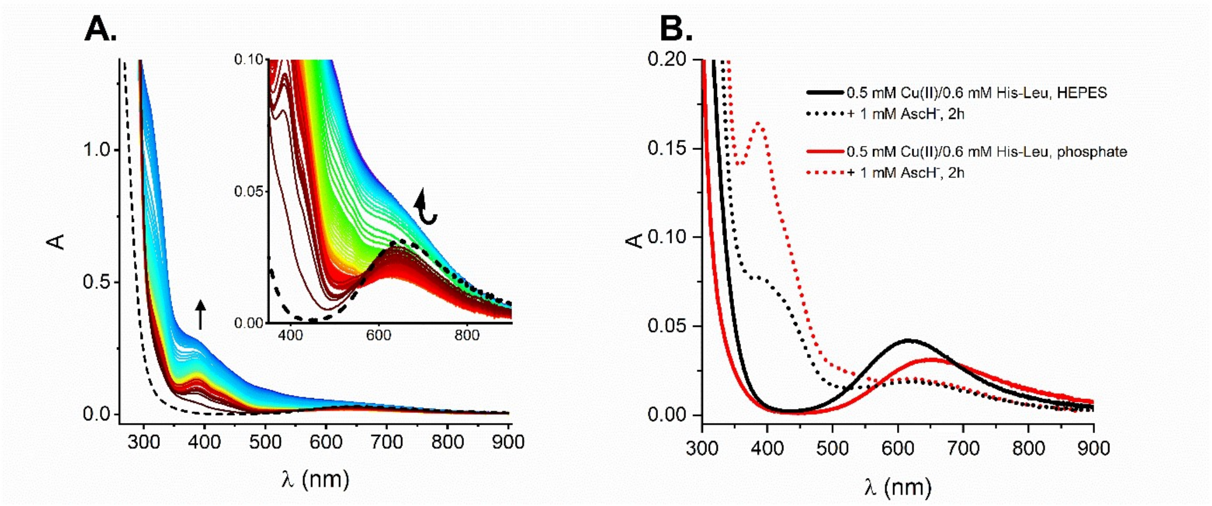

Figure S15. (A) UV-vis spectra recorded during the incubation of 0.5 mM Cu(II)/0.6 mM His-Leu with 1 mM AscH$^-$ in 50 mM phosphate buffer pH 7.4 for 24 h. The dashed spectrum refers to signals for Cu(II)/His-Leu complexes in phosphate without AscH$^-$. The spectra after the AscH$^-$ addition are codded with rainbow colors from dark red to blue. The insets are for zooming the changes near the d-d and region. The arrows indicate the direction of the band changes during the reaction. (B) The comparison of the UV-vis spectra of 0.5 mM Cu(II)/0.6 mM His-Leu in 50 mM HEPES pH 7.4 (solid lines) and in 50 mM phosphates (dotted lines) without ascorbate (black lines) and after 2-hour incubation with ascorbate (red lines).

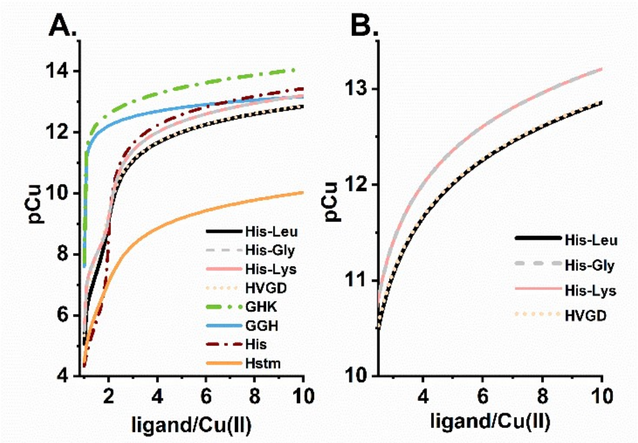

Figure S16. The comparison of Cu(II) affinity towards selected ligands given as pCu calculated based on the potentiometric constants (His-Leu − this study, His-Gly,[3] His-Lys,[4] HVGD,[5,6] GHK,[7] GGH,[8] histidine His,[1] histamine Hstm,[2] for 1 mM Cu(II) and 1–10 mM ligand concentrations at pH 7.4 (A). For clarity, the curves for the His−1 peptides are shown separately for the ligand/ Cu(II) molar ratio range 2.5 – 10 (B).

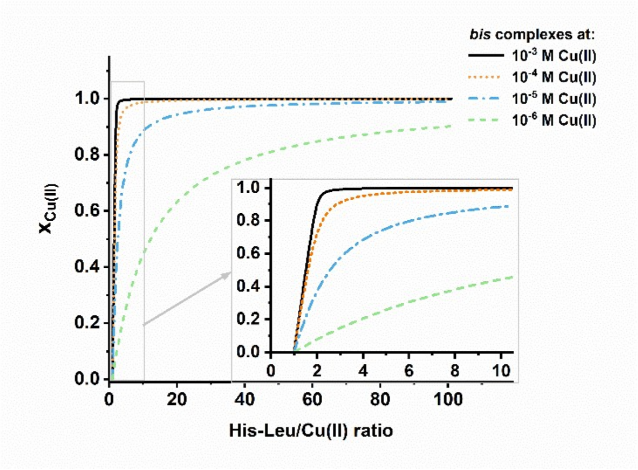

Figure S17. Cu(II) molar fraction for *bis* complexes of His-Leu and Cu(II) ions calculated based on the potentiometric constants for the His−Leu/Cu(II) molar ratio range 1−100 and the Cu(II) concentrations from 1 µM to 1 mM at pH 7.4. For clarity, the inset represents the curves in the narrowed His-Leu/Cu(II) molar ratio range 1−10.

References

(1) Freeman, H. C.; Martin, R. P. Potentiometric Study of Equilibria in Aqueous Solution between Copper(II) Ions, L (or D)-Histidine and L-Threonine and Their Mixtures. *Journal of Biological Chemistry* **1969**, *244* (18), 4823–4830. https://doi.org/10.1016/s0021-9258(18)94278-x.

(2) Török, I.; Gajda, T.; Gyurcsik, B.; Tóth, G. K.; Péter, A. Metal Complexes of Imidazole Ligands Containing Histamine-like Donor Sets: Equilibrium, Solution Structure and Hydrolytic Activity. *J.Chem.Soc.,Dalton Trans.* **1998**, 1205–1212. https://doi.org/10.1039/A707408E.

(3) Sóvágó, I.; Farkas, E.; Gergely, A. Studies on Transition-Metal-Peptide Complexes. Part 7. Copper(II) Complexes of Dipeptides Containing L-Histidine. *Journal of the Chemical Society, Dalton Transactions* **1982**, 2159-2163. https://doi.org/10.1039/DT9820002159.

(4) Remelli, M.; Conato, C.; Agarossi, A.; Pulidori, F.; Koz, H. Copper Complexes of Dipeptides with L-Lys as C-Terminal Residue: A Thermodynamic and Spectroscopic Study. *Polyhedron* **2000**, *19*, 2409–2419. https://doi.org/10.1016/S0277-5387(00)00539-8.

(5) Myari, A.; Malandrinos, G.; Plakatouras, J.; Hadjiliadis, N.; Sóvágó, I. Interaction of Cu(II) with His-Val-Gly-Asp and of Zn(II) with His-Val-His, Two Peptides at the Active Site of Cu,Zn-Superoxide Dismutase. *Bioinorg Chem Appl* **2003**, *1* (1), 99–112. https://doi.org/10.1155/S1565363303000086.

(6) Myari, A.; Malandrinos, G.; Deligiannakis, Y.; Plakatouras, J. C.; Hadjiliadis, N.; Nagy, Z.; Sovago, I. Interaction of Cu with His–Val–His and of Zn with His–Val–Gly–Asp, Two Peptides Surrounding Metal Ions in Cu,Zn-Superoxide Dismutase Enzyme. *J Inorg Biochem* **2001**, *85*, 253–261. https://doi.org/10.1016/s0162-0134(01)00204-5.

(7) Bossak-Ahmad, K.; Bal, W.; Frączyk, T.; Drew, S. C. Ternary Cu^{2+} Complexes of Human Serum Albumin and Glycyl- (L)-Histidyl- (L)-Lysine. *Inorg Chem* **2021**, 60 (22), 16927–16931. https://doi.org/10.1021/acs.inorgchem.1c03084.

(8) Bossak-Ahmad, K.; Frączyk, T.; Bal, W.; Drew, S. The Sub-picomolar Cu^{2+} Affinity of Human Serum Albumin. *ChemBioChem* **2019**, *21* (3), 331–334. https://doi.org/10.1002/cbic.201900435.
